# Chronotherapy in head and neck cancer: A systematic review and meta‐analysis

**DOI:** 10.1002/ijc.35234

**Published:** 2024-11-07

**Authors:** Mohammad Abusamak, Abdel‐Azez Abu‐Samak, Wenji Cai, Haider Al‐Waeli, Faez Saleh Al‐Hamed, Mohammad Al‐Tamimi, Malik Juweid, Akhilanand Chaurasia, Belinda Nicolau, Faleh Tamimi

**Affiliations:** ^1^ Faculty of Dental Medicine and Oral Health Sciences McGill University Montreal Quebec Canada; ^2^ Faculty of Dentistry Dalhousie University Halifax Nova Scotia Canada; ^3^ Rutgers‐Trinitas Regional Medical Center Elizabeth New Jersey USA; ^4^ College of Dental Medicine, QU Health Qatar University Doha Qatar; ^5^ Faculty of Dentistry University of Toronto Toronto Ontario Canada; ^6^ Department of Radiology and Nuclear Medicine, School of Medicine University of Jordan Amman Jordan; ^7^ Department of Oral Medicine and Radiology King George's Medical University Lucknow India

**Keywords:** chemotherapy, chronotherapy, circadian rhythm, head and neck cancer, radiotherapy

## Abstract

Optimizing the timing of radiotherapy and chemotherapy tailored to the body's biological clock (i.e., chronotherapy) might improve treatment efficacy and reduce side effects. This systematic review evaluated the effect of chrono‐radiotherapy and chrono‐chemotherapy on treatment efficacy, toxicity and adverse events in head and neck cancer (HNC) patients from prospective and retrospective studies published between the date of database inception until March 2024. The primary outcome measures for chrono‐radiotherapy were treatment efficacy and incidence of grade ≥3 oral mucositis, and the main outcome measures for chrono‐chemotherapy were objective response rate (ORR) and overall toxicity and adverse events. Of 7349 records identified, 22 studies with 3366 patients were included (chrono‐radiotherapy = 9 and chrono‐chemotherapy = 13). HNC patients who underwent chrono‐radiotherapy had 31% less risk of developing severe oral mucositis (grade ≥3) compared to evening radiotherapy (risk ratio: 0.69, 95% CI: 0.53–0.90, *p* < 0.05). Further, HNC patients who underwent chrono‐chemotherapy using platinum‐based and antimetabolite agents had 73% less risk of lower ORR compared to nontime‐stipulated chemotherapy (risk ratio: 0.27, 95% CI: 0.09–0.84, *p* < 0.05). In addition, HNC patients who underwent chrono‐chemotherapy had 41% less risk of lower overall toxicity and adverse events in comparison to nontime‐stipulated chemotherapy (risk ratio: 0.59, 95% CI: 0.47–0.72, *p* < 0.05). In conclusion, chrono‐chemotherapy studies showed evidence of improved treatment efficacy, while in chrono‐radiotherapy it was maintained. Chrono‐radiotherapy and chrono‐chemotherapy studies provide evidence of reduced toxicity and adverse events. However, optimized circadian‐based multicentric clinical studies are needed to support chrono‐radiotherapy and chrono‐chemotherapy in managing HNC.

## What's New?

Adverse events are common after chemotherapy and radiotherapy. Chronotherapy, which tailors treatment to the circadian rhythm, aims to reduce the frequency of adverse events and improve treatment efficacy. Here, the authors conducted a systematic review of chrono‐chemotherapy and chrono‐radiotherapy in patients with head and neck cancer (HNC). Their analysis included 22 studies and 3366 patients. Patients who received chrono‐radiotherapy had fewer instances of oral mucositis than those who received evening radiotherapy, they found; patients who received chrono‐chemotherapy experienced lower overall toxicity and improved objective response rate than those who received nontime‐stipulated chemotherapy.

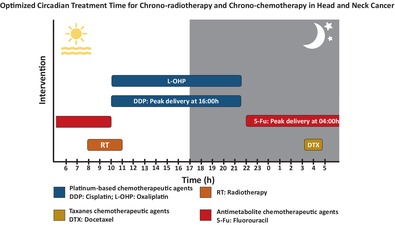

## 1 INTRODUCTION

Head and neck cancer (HNC) was the sixteenth most common cancer globally in 2022, accounting annually for 4.7% of new cases and 4.9% of deaths.^1^ In fact, the incidence of new cases is predicted to increase by 30% in 2030, that is approximately 1 million new cases annually.^2^ The primary risk factors include heavy alcohol intake, tobacco use, oncogenic viruses such as the human papilloma virus (oropharyngeal) and Epstein–Barr virus (nasopharyngeal cancer).^3^ HNC is staged according to the Tumor, Node and Metastasis (TNM) staging system. Stages I and II are early‐stage cancer, while stages III and IV are locally advanced and recurrent or metastatic cancers.^3^, ^4^ Treatment modalities for HNC are comprised of (1) surgery, (2) radiotherapy and (3) systemic therapies, such as chemotherapy, targeted therapy and immunotherapy.^3^, ^4^

As the cancer stage advances, treatment modalities are often comprised of numerous treatment combinations. For instance, early‐stage cancers are managed by a single modality (surgery or radiotherapy).^5^ On the other hand, locally advanced cancers are managed by multiple treatment modalities: (1) either surgery followed by chemo‐radiotherapy or adjunct radiotherapy, or (2) definitive chemo‐radiotherapy without surgery.^5^ Although treatment modalities often overlap, treatments remain mainly specific to cancer site and histopathological stages,^3^ in addition to several factors that may affect the survival and prognosis namely: cancer stage, site involved and oncogenic virus status.^6^ For example, HNC stages I‐II were found to have a 70%–90% 5‐year overall survival, in comparison with 40% 5‐year overall survival rate found in stages III‐IV.^6^ Indeed, there has been limited improvement in HNC survival rates over the last three decades (5‐year survival rate 55% to 66%).^7^ HNC has high treatment cost with a poor survival rate and increased morbidity after treatment and is considered one of the most impairing human diseases.^8^ Also, HNC survivors have the second highest suicide rates compared to other cancers, and is about four times higher than the US general population.^9^

Adverse events (short‐term and long‐term) are common after chemotherapy and radiotherapy in HNC treatments. These events include oral mucositis, xerostomia, dermatitis and hematological and gastrointestinal toxicities.^10^ While some of the adverse events may be inevitable, minimizing their severity is paramount to prevent treatment interruption that negatively affects clinical outcomes.^11^ One approach that has been developed to minimize adverse events is intensity‐modulated radiation therapy (IMRT). IMRT aims to reduce radiation‐induced toxicities by limiting radiation dose to critical structures only (e.g., nerves).^12^ Consequently, IMRT may increase the total radiation dose to other unshielded structures.^13^ Another novel radiation approach is the proton beam therapy that utilizes the so called ‘Bragg peak’ phenomenon to deposit most of the proton energy at their target, while reducing the residual radiation dose at the healthy tissues.^14^ However, further research in terms of cost considerations and limited availability associated with proton therapy is still needed.^14^ In contrast to radiotherapy, there are two options for chemotherapeutic medications when the dose‐limiting toxicity is reached; either the treatment must be discontinued or dose reduced.^15^

Chronotherapy is an emerging field that investigates dosing time of medical interventions, tailored to the circadian rhythm. Chronotherapy of radiotherapy (chrono‐radiotherapy) aims to improve treatment efficacy and/or reduce radiation induced adverse events based on two concepts. First, the circadian clock and cell cycle are tightly linked and their circadian fluctuations are at the same frequency.^16^ In fact, the circadian clock genes such as BMAL1, CLOCK, PER and CRY regulate directly/indirectly various genes and proteins that are key transition points on the cell cycle namely c‐Myc and Cyclinc‐D1, Cyclin E, Cyclin A, Cyclinc‐B1, p27 and p53.^16^ In addition, the cell cycle proteins have different expression levels with peak expression of p27 (early G_1_ phase marker) at 06:00 h, p53 (late G_1_ phase marker) at 10:50 h, cyclin E (S phase marker) at 14:50 h, cyclin‐A (G_2_ phase marker) at 16:00 h and Cyclin‐B1 (G_2_/M phase marker) at 21:10 h.^17^, ^18^ The evidence shows that the expression of PER1 coincides with p53 in the morning, while BMAL1 is synchronized with Cyclin B1 peaking at night.^16^ Furthermore, cells during different cell cycle phases exhibited different sensitivity towards radiation with cells being most radiosensitive in G_2_/M phase, less sensitive in G_1_ phase and least sensitive in late S phase.^18^ Interestingly, cells in G_2_/M phase were reported to be at least two times as many cells in the afternoon and cells in G_1_ phase at least twice as many in the morning.^18^ The second concept relies on the fact that cancer cells are dysthymic and their circadian clock is disrupted and deregulated, whilst healthy cells exhibit a robust circadian rhythm.^19^ Therefore, when combing these two concepts together, radiotherapy in the morning (G_1_ phase) would be the least susceptible to radiation‐induced adverse events in normal tissues compared to evening or nighttime radiotherapy.

The success of chronotherapy of chemotherapy (chrono‐chemotherapy) could be attributed to the fact that both pharmacodynamics and pharmacokinetics, similar to major biological functions, are subjected to circadian changes.^20^ Pharmacokinetics aims to provide an ideal drug concentration that is a balance between efficacy and toxicity. There are four phases included in the pharmacokinetic process namely drug absorption, distribution, metabolism and excretion (ADME).^20^ These four phases exhibit circadian variation and express various transporters and drug‐metabolizing enzymes. For example, the expression of Cyp3a11, a drug metabolizing enzyme in the liver, is regulated by BMAL1.^21^ Evidence showed that the circadian system not only modulates chemotherapeutic agents through drug metabolism and detoxification, but it can also regulate integral molecular processes such as cell cycle, and DNA repair and apoptosis essential to successful anti‐cancer treatment.^22^, ^23^ To date, the efficacy and toxicity of more than 15 chemotherapeutic agents used for cancer treatment showed time‐dependency.^22^, ^23^ The optimum time for platinum‐based chemotherapeutic agents (cisplatin, carboplatin and oxaliplatin) is reported to be in the evening with a peak delivery at 16:00 h, which coincides with an antioxidant molecule that prevents cell damage and toxicities caused by these drugs called Glutathione (GSH) that also peaks at 16:00 h.^20^ In addition, the efficacy and toxicity of Fluorouracil depend on the oscillation in its rate limiting degrading enzyme, dihydropyrimidine dehydrogenase (DPD) and therapeutic target, thymidylate synthase (TS).^20^ They both show diurnal variations with DPD peaking at 01:00 h and TS at 17:00 h.^24^ On the other hand, Taxanes (paclitaxel and docetaxel) seemed to be more tolerated in rodents in the resting phase, which corresponds to nighttime administration in cancer patients.^25^ The above‐mentioned preclinical evidence outlines the bases of chronotherapeutic scheduling in chemotherapy medications to reduce toxicity and adverse events in cancer treatments.

Nevertheless, chrono‐radiotherapy and chrono‐chemotherapy have shown promising results in treating various cancers, such as lung, cervical and colorectal cancers.^26^, ^27^, ^28^, ^29^ Specifically, evidence shows that chronotherapy could reduce the severity of adverse events such as oral mucositis and hematological toxicities.^26^, ^27^, ^28^, ^29^ However, the efficacy of chronotherapy in treating cancer is inconsistently reported.^26^, ^27^, ^28^, ^29^ In addition, our recent scoping review reported favourable outcomes of chronotherapy in HNC, which highlighted the need for further investigation via a systematic review and meta‐analysis.^30^ Therefore, this systematic review aimed to evaluate the clinical evidence of the effect of chrono‐radiotherapy and chrono‐chemotherapy on the treatments' efficacy, toxicity and adverse events in HNC.

## 2 METHODS

This systematic review and meta‐analysis is reported according to the Preferred Reporting Items for Systematic Reviews and Meta‐Analyses (PRISMA) guidelines.^31^ Relevant protocol was registered on the International Prospective Register of Systematic Reviews (PROSPERO—reference number: CRD42022295964).

### 2.1 Objectives and research questions

This systematic review and meta‐analysis had two objectives. First, to evaluate the efficacy and toxicity of chrono‐radiotherapy in the management of HNC. Second, to evaluate the efficacy of and toxicity of chrono‐chemotherapy in treating HNC. Our research questions for each objective were as follows:

1‐ Among HNC patients undergoing radiotherapy, does chronotherapy, in comparison to nontime‐stipulated radiotherapy, improve therapeutic efficacy and/or reduce toxicity and adverse effects?
2‐ Among HNC patients undergoing chemotherapy, does chronotherapy, in comparison to nontime‐stipulated chemotherapy, improve treatment efficacy and/or reduce toxicity and adverse effects?

### 2.2 Search strategy and selection criteria

A trained medical librarian (MM) conducted a systematic search using Medical Subject Headings (MeSH), keywords and Boolean operators (OR/AND) in four online databases (MEDLINE, Embase, CINAHL and Scopus) from the date of inception (Table S1). Searches were carried out on 29 June 2022, and then updated on 11 March 2024. The screening was limited to articles written in English and Chinese to match our team's expertise.

**FIGURE 1:**
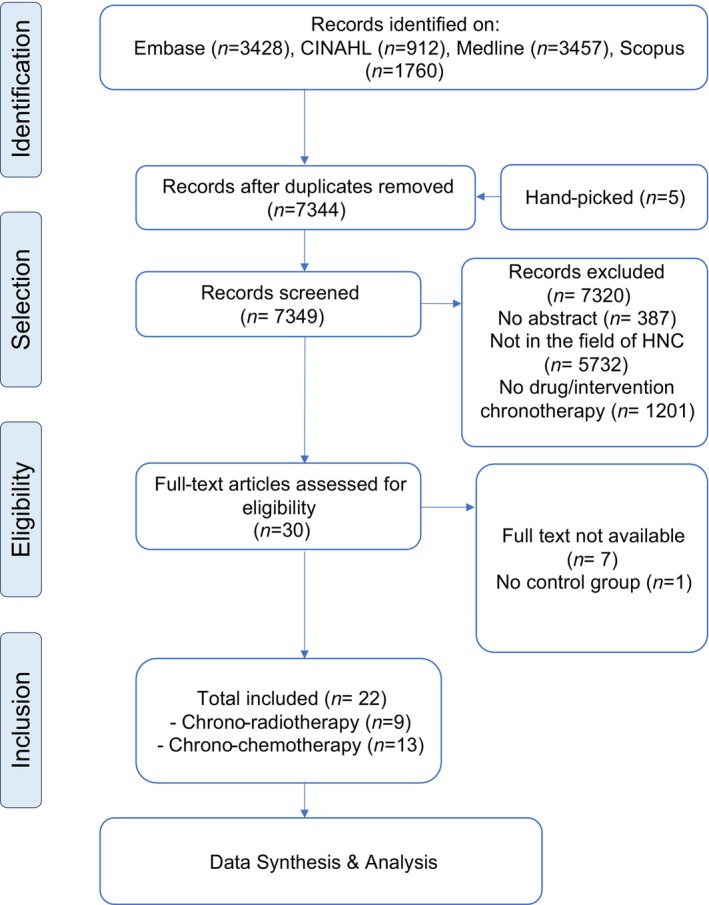
PRISMA flow chart of included studies.

The article selection process is outlined in a PRISMA diagram (Figure 1). After references were exported from the online databases, all duplicates were removed by EndNote X9 citation management software. Next, two blinded independent reviewers (MA & AA) screened the articles on Rayyan by title/abstract first, then by full‐text if available. Disagreements between reviewers were resolved by a third reviewer (MT). A Cohen kappa score of 0.782 was calculated showing substantial agreement between the reviewers.

The review only included original experimental and observational studies investigating chronotherapy of radiotherapy and/or chemotherapy in HNC adult (≥18 years) patients. In addition, only randomized clinical trials (RCTs) were included in the meta‐analysis. Otherwise, records with no abstracts, not in the field of HNC, that did not investigate chronotherapy (timed interventions), or with no control group for comparisons were excluded.

### 2.3 Data charting and data synthesis

Two reviewers (MA & WC) extracted and charted the data from included studies using Excel sheets and then the extracted data were summarized comprising each study's authors, title, publication year, study design, number of participants, age, sex, tumour type, site, intervention, control, outcome and results. The same two reviewers also independently critically appraised the quality of included papers using the Joanna Briggs Institute (JBI) risk of bias assessment tool for each study design appropriately.^32^

Treatment efficacy of chrono‐radiotherapy and chrono‐chemotherapy was measured according to World Health Organization (WHO) and Response Evaluation Criteria in Solid Tumours (RECIST) for disease response. On the other hand, toxicity (early and late) and adverse events secondary to HNC treatment involving one or more of the gastrointestinal, haematological, neurological and dermatological systems were measured using National Cancer Institute of Canada Common Toxicity Criteria (NCIC CTC), Radiation Therapy Oncology Group (RTOG), Common Terminology Criteria for Adverse Events (CTCAE) and WHO criteria.

### 2.4 Statistical analysis

The primary comparison in chrono‐radiotherapy studies was the incidence of severe oral mucositis (grade ≥3). Treatment efficacy could not be analyzed due to dissimilar end‐point measurements between studies. In chrono‐chemotherapy studies, the primary comparison was objective response rate (ORR) and toxicities and adverse events (grade ≥3). Toxicities and adverse events were not analyzed if both groups had zero events or endpoint measure was reported in less than three studies. We used the Mantel‐Haenszel statistical method (without continuity correction) with a fixed‐effect model for chrono‐radiotherapy studies and a random‐effects model for chrono‐chemotherapy studies.^33^ Risk ratio (RR) and its 95% confidence interval (CI) was calculated to measure the effect size of our outcomes. Paule‐Mandel estimator was used to calculate the heterogeneity variance τ^2^, and Knapp‐Hartung adjustments to calculate the CI around pooled effect.^34^, ^35^ Also, Higgins and Thompson's *Ι* ^2^ and Cochran's *Q* tests were performed to assess heterogeneity among included studies (*χ* ^2^ = *p* > 0.10; *I* ^2^ < 25%).^36^, ^37^ Further, prediction interval was calculated to present the intervention effects that could be evident in future studies. Publication bias was assessed using funnel plots, then Trim and Fill method was used for asymmetry adjustment if the number of included studies in the analysis was more than 10. Finally, R Studio, version 4.2.3 was used to perform all analyses (namely meta^38^ and metafor^39^ packages) and create forest plot figures.

## 3 RESULTS

A total of 7349 records were screened by title and abstract, and 7320 were excluded as they did not meet the inclusion criteria. Next, seven articles were excluded as their full texts were not available and one article was excluded due to the absence of control group for comparisons. Finally, 22 studies were included in this systematic review (chrono‐radiotherapy = 9,^40^, ^41^, ^42^, ^43^, ^44^, ^45^, ^46^, ^47^, ^48^ and chrono‐chemotherapy = 13^49^, ^50^, ^51^, ^52^, ^53^, ^54^, ^55^, ^56^, ^57^, ^58^, ^59^, ^60^, ^61^). The overall quality of included studies ranged from fair (13 out of 22) to good (nine out of 22) (Table S2). Both chrono‐radiotherapy and chrono‐chemotherapy RCTs had high risk of bias due to absence of blinded treatment assignment and/or to outcome assessments.

### 3.1 Chrono‐radiotherapy

#### 3.1.1 Characteristics, study designs and interventions

Of the nine studies investigating the efficacy of chrono‐radiotherapy in HNC, three studies were RCTs (*n* = 492 patients), two were non‐RCTs (*n* = 284 patients) and four were retrospective cohorts (*n* = 1604 patients). Overall, included papers were heterogenous in terms of study design, intervention and population. In addition, the majority of patients were males, aged between 35 and 65 years, diagnosed with HNC squamous cell carcinomas and underwent concurrent chemotherapy. Furthermore, different radiotherapy techniques with variable irradiation doses ranging from 50 to 70 Gy were used. The intervention duration was different among studies but similar between groups within each study. Finally, only three studies reported their sample size calculations, and the power of the remaining six studies could not be calculated. (Table 1, Table S3).

**TABLE 1:** Demographics and characteristics of chrono‐radiotherapy included studies.

Author/Year (Country)	*n*	Age (years)	Sex (M vs. F)	Site^a^	Concurrent chemotherapy (% of patient)
Randomized clinical trials (1:1)					
Bjarnason et al. 2009 (Canada)	216	Median = 60	77% vs. 23%	Oral cavity (19%)	No
				Oropharynx (35%)	
				Hypopharynx (5%)	
				Nasopharynx (1%)	
				Supraglottic larynx (7%)	
				Glottic larynx (2%)	
				Subglottic larynx (1%)	
				Left neck nodes (22%)	
				Right neck nodes (23%)	
Goyal et al. 2009 (India)	212	<35 (12.9%)	81% vs. 19%	Tongue (15%)	No
		35–50 (50.8%)			
				Gingiva (24%)	
				Mouth floor (16%)	
		50–70 (36.3%)			
				Retromolar trigone (10%)	
				Lips (10%)	
				Pharynx (12%)	
				Larynx (12%)	
Lavanya and Arulponni 2021 (India)	64	Mean = 56	72% vs. 28%	Oral cavity (26.5%)	Yes (100%)
				Oropharynx (20.5%)	
				Hypopharynx (31%)	
				Larynx (22%)	
Nonrandomized clinical trials					
Elzahi et al. 2020 (Egypt)	160	Mean = 47.12	63% vs. 37%	Nasopharynx (26.8%)	Yes (83%)
				Oropharynx (15.7%)	
				Larynx (30.6%)	
				Hypopharynx (5.65%)	
				Salivary gland (9.4%)	
				Oral cavity (11.85%)	
Ponna et al. 2021 (India)	124	Median = 54	68% vs. 32%	Nasopharynx (37%)	No
				Oral cavity (27%)	
				Hypopharynx (14%)	
				Others (22%)	
Retrospective cohorts					
Kuriakose et al. 2016 (India)	142	Mean = 56.11	92% vs. 8%	Oral cavity (40.1%)	Yes (53.5%)
				Oropharynx (52.8%)	
				Nasopharynx (7.1%)	
Gu et al. 2020 (United States)	190	Mean = 61.8	78% vs. 22%	Larynx (29%)	Yes (84.2%)
				Lip oral (13.7%)	
				Pharynx (47.4%)	
				Others (10%)	
Brolese et al. 2021 (Switzerland)	617	Median = 62	77% vs. 23%	Oral cavity (17.7%)	Yes (85.3%)
				Oropharynx (45.4%)	
				Larynx (16%)	
				Hypopharynx (13.8%)	
				Others (7.1%)	
Elicin et al. 2021 (Switzerland)	655	Median = 65	77% vs. 23%	Oral Cavity (11.9%)	Yes (80.6%)
				Oropharynx (45%)	
				Larynx (22.3%)	
				Hypopharynx (13.9%)	
				Others (6.9%)	

^a^ Majority are HNC squamous cell carcinoma.

The three RCTs assigned patients into morning (chrono‐radiotherapy) and evening groups^40^, ^41^, ^42^ (Table 2). Chrono‐radiotherapy groups were treated from 08:00 h to 10:00 h except for Bjarnason et al. (2009), who treated their patients from 08:00 h to 11:00 h (1 h extra). On the other hand, evening groups were either treated from 16:00 h to 18:00 h, 15:00 h to 18:00 h or 17:00 h to 20:00 h. The included non‐RCTs also divided patients into two groups with 2 h intervals.^43^, ^44^ Elzahi et al. (2020) irradiated the chrono‐radiotherapy group between 06:00 h and 08:00 h and the evening group between 13:00 h and 15:00 h,^43^ while Ponna et al. (2021) irradiated the chrono‐radiotherapy and evening groups between 08:00 h to 10:00 h and 15:00 h and 17:00 h,^44^ respectively.

**TABLE 2:** Chrono‐radiotherapy toxicity and adverse events.

Author/Year	Treatment Time				Irradiation dose	End‐points	Significant difference (favouring MRT; *p* < 0.05)
	Morning RT	*n*	Evening RT	*n*			
Randomized clinical trials (1:1)							
Bjarnason et al. 2009	08:00 h–10:00 h	104	16:00 h–18:00 h	101	50–70 Gy in 25–35 fractions	Incidence of grade ≥ 3 oral mucositis^a^	Yes
						Interval to the development of Grade ≥2^a^	Yes
						Duration of various grades of oral mucositis	No
						Proportion of patients with ≥1 Tx days lost because of toxicity	No
						Incidence of other acute and late toxicities	No
Goyal et al. 2009	08:00 h–11:00 h	88	15:00 h–18:00 h	89	≥ 60 Gy	Incidence of oral mucositis grade ≥3	No
						Progression rate of oral mucositis—7 weeks	Yes. Week 4 & 7
						Incidence of skin reaction	No
Lavanya and Arulponni 2021	08:00 h–11:00 h	32	17:00 h–20:00 h	32	66 Gy in 30 fractions	Incidence of oral mucositis	No
						Weight loss	No
Nonrandomized clinical trials							
Elzahi et al. 2020	06:00 h–08:00 h	80	13:00 h–15:00 h	80	65–70 Gy	Mouth and throat soreness severity	Yes
Ponna et al. 2021	08:00 h–10:00 h	62	15:00 h–17:00 h	62	66–70 Gy in 30–35 fractions	Onset of oral mucositis	Yes
						Incidence of oral mucositis grade ≥ 3	Yes
						Median time to develop severe oral mucositis	Yes
Retrospective cohorts							
Kuriakose et al. 2016	08:00 h–11:00 h	73	17:00 h–20:00 h	69	60–66 Gy in 30–33 fractions	Incidence of oral mucositis grade ≥ 3	Yes
						RT interruptions due to toxicity	Yes
						Mean time to develop oral mucositis grade ≥ 3	No
Gu et al. 2020	08:30 h−09:30 h	32	12:30 h–14:00 h	25	56–70 Gy in 35 fractions	Average treatment timing and repeated mouth throat soreness measures	Yes
	09:30 h–10:30 h	32	14:00 h–15:00 h	25			
	10:30 h–11:30 h	36	15:00 h–16:30 h	14			
						Maximum mouth throat soreness	Yes
	11:30 h −12:30 h	26					
						Incidence of severe oral mucositis scores ≥3	Yes
Brolese et al. 2021	00:00 h–12:00 h	336	12:00 h–00:00 h	28	≥ 60 Gy in 2 Gy daily fraction	Mean acute toxicity scores	Yes
						Mean late toxicity scores	No

Abbreviation: RT, radiotherapy.

^a^ Only in subgroup received ≥66 Gy of radiation.

Regarding the retrospective cohorts, only one study divided participants into two groups treated between 08:00 h to 11:00 h and 17:00 h to 20:00 h for chrono‐radiotherapy and evening groups, respectively.^45^ Two studies dichotomized patients into AM (00:00 h–12:00 h) and PM (12:00 h–00:00 h) with an approximate irradiation duration of 2 h.^47^, ^48^ Finally, the last cohort study divided their patients into seven groups, with 1 h–1.5 h radiotherapy sessions for each group between 08:30 h and 16:30 h.^46^

#### 3.1.2 Treatment efficacy

Four included studies investigated treatment efficacy of chrono‐radiotherapy (morning radiotherapy) and evening radiotherapy.^40^, ^41^, ^44^, ^48^ There were no significant differences reported between groups in terms of treatment efficacy (Table S4).

#### 3.1.3 Toxicity and adverse events

Eight out of nine studies evaluated toxicity and adverse events (Table 2). All chrono‐radiotherapy (morning radiotherapy) groups showed fewer side effects when compared to evening radiotherapy groups in treating HNC patients. Only two out of three RCTs reported significant findings.^40^, ^41^ Bjarnason et al. (2009) found significant reduction in the incidence of oral mucositis grade ≥3 and longer interval to develop oral mucositis grade ≥2 in chrono‐radiotherapy compared to evening radiotherapy only in a subgroup of patients receiving ≥66 Gy of radiation.^40^ Goyal et al. (2009) evaluated oral mucositis progression for 7 weeks and reported significant slower progression at weeks 4 and 7 in the chrono‐radiotherapy group.^41^ On the other hand, both non‐RCTs and two retrospective cohorts demonstrated significant reduction in oral mucositis severity and incidence in the chrono‐radiotherapy group^43^, ^44^, ^45^, ^46^ (Table 2, Table S5).

#### 3.1.4 Chrono‐radiotherapy effect on oral mucositis severity (Grade ≥3)

**FIGURE 2:**
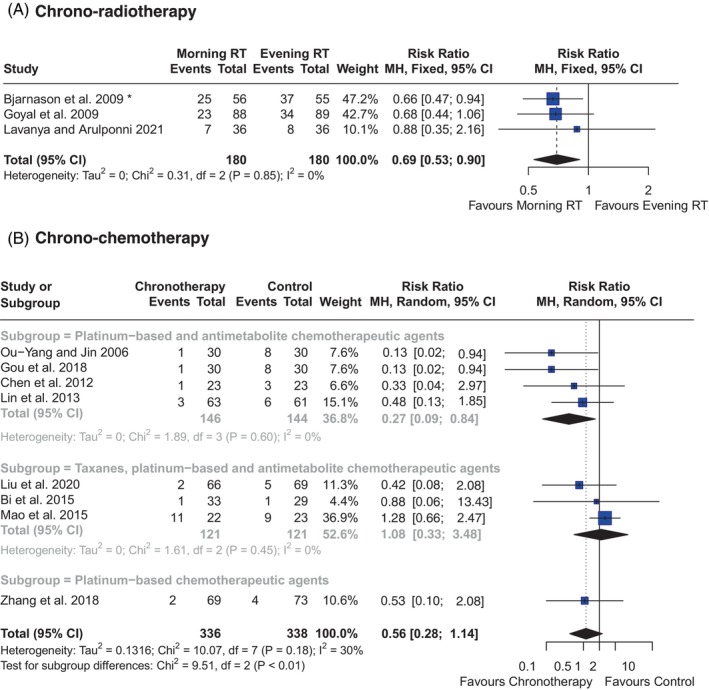
(A) Effect of chrono‐radiotherapy (morning) on oral mucositis severity (Grade 3). RT, Radiotherapy. * Subgroup that received ≥66 Gy of radiation. (B) Effect of chrono‐chemotherapy on objective response rate.

The incidence of oral mucositis was reported in all included chrono‐radiotherapy RCTs (*n* = 3). The estimated variance of between‐study heterogeneity was *τ* ^2^ = 0 (95% CI: 0.00–0.75), with an *Ι* ^2^ value of 0% (95% CI: 0.0%–98.6%), and the prediction interval ranged between 0.127 and 3.10. As shown in Figure 2A, HNC patients who underwent chrono‐radiotherapy (morning) had 31% less risk of developing severe oral mucositis (grade ≥3) compared to patients prescribed evening radiotherapy (RR: 0.69*, 95% CI: 0.53–0.90, *p* < 0.05). * = The subgroup that received ≥66 Gy of radiation from Bjarnason et al. (2009) was used for pool effect size estimate.

### 3.2 Chrono‐chemotherapy

#### 3.2.1 Characteristics, study designs and interventions

Of the 13 included studies investigating the efficacy of chrono‐chemotherapy in HNC, 11 were RCTs (*n* = 787) and two were retrospective cohorts (*n* = 199). The main characteristics of included studies are presented in Table 3. Overall, included studies were heterogenous in terms of study design, intervention (dose, regimen and duration) and population, and the majority of patients were male, aged between 50 and 60 years and diagnosed with nasopharyngeal carcinoma (66%). Tumour stages varied between I and IVc. Only one retrospective study recruited patient diagnosed with recurrent and/or metastatic HNC squamous cell carcinomas. All patients in the included studies, except one, underwent nontime‐stipulated radiotherapy (Table 3, Table S6). Furthermore, three studies used platinum‐based agents (i.e., cisplatin) only,^52^, ^55^, ^61^ while four studies used a combination of platinum‐based and antimetabolite agents,^49^, ^50^, ^51^, ^58^ and six studies used taxanes, platinum‐based and antimetabolite chemotherapeutic agents.^53^, ^54^, ^56^, ^57^, ^59^, ^60^

**TABLE 3:** Demographics and characteristics of chrono‐chemotherapy included studies.

Author/Year (Country)	*n*	Age (years)	Sex (M vs. F)	Tumour/Site (Stage)	Comparators				
					Drug	Intervention	*n*	Control	*n*
Randomized clinical trials (1:1)									
Ou‐Yang and Jin 2006 (China)	60	Median = 54.5	65% vs. 35%	NPC (I‐IV) [Unspecified Criteria]	Cisplatin	10:00 h–22:00 h	30	10:00 h, duration unspecified	30
					Fluorouracil	22:00 h–10:00 h		10:00 h, duration unspecified	
Chen et al. 2012 (China)	46	Median = 43.8	74% vs. 26%	NPC (UICC 2002 stage II‐IV)	Oxaliplatin	10:00 h–22:00 h	23	9:00 h, duration unspecified	23
					Fluorouracil	22:00 h–10:00 h		9:00 h, duration unspecified	
Lin et al. 2013 (China)	125	Median = 42	79% vs. 21%	NPC (WHO II & III)	Cisplatin	10:00 h–22:00 h with peak delivery at 16:00 h	63	10:00 h–22:00 h	61
					Fluorouracil	22:00 h–10:00 h with peak delivery at 04:00 h		22:00 h–10:00 h	
Verma et al. 2014 (India)	60	31–40 (16.5%)	95% vs. 5%	LAHNSCC (AJCC 2010 III & IVB)	Cisplatin	18:00 h, duration unspecified	30	06:00 h, duration unspecified	30
		41–50 (25.0%)							
		51–60 (35.0%)							
		61–70 (23.5%)							
Bi et al. 2015 (China)	66	Median = 45.9	70% vs. 30%	NPC (UICC 2010 stage III‐IVb)	Docetaxel	03: 30 h–04: 30 h	36	Time and duration unspecified	30
					Cisplatin	10:00 h–22:00 h		Time and duration unspecified	
					Fluorouracil	22:00 h–10:00 h		Time and duration unspecified	
Mao et al. 2015 (China)	46	Median = 48	78% vs. 22%	NPC (UICC 2010 stage IVc)	Docetaxel	06/07:00 h–10:00 h	22	Time and duration unspecified	23
					Cisplatin	10:00 h–22:00 h		Time and duration unspecified	
					Fluorouracil	22:00 h–10:00 h		Time and duration unspecified	
Zhang et al. 2018 (China)	148	Median = 46	70% vs. 30%	NPC (2010 UICC IIIa‐IVb)	Cisplatin	10:00 h–22:00 h with peak delivery at 16:00 h	74	10:00 h–14:00 h	74
Li et al. 2018 (China)	32	Mean = 51.8	63% vs. 37%	OSCC (III & IV)	Docetaxel	18:30 h–19:30 h	16	1 h after breakfast, duration unspecified	16
					Cisplatin	19:30 h −21:30 h		2 h after breakfast, duration unspecified	
					Fluorouracil	10:00 h–18:00 h		8 h duration, time unspecified	
Liu et al. 2020 (China)	135	51.1% (> 46)	73% vs. 27%	NPC (stage III‐IVb)	Docetaxel	03:30 h–04:30 h	66	10:00 h, duration unspecified	69
		48.9% (≤ 46)			Cisplatin	10:00 h–22:00 h		10:00 h, duration unspecified	
					Fluorouracil	22:00 h–10:00 h		10:00 h, 24 h infusion.	
Gou et al. 2021 (China)	60	Median = 48.5	78% vs. 22%	NPC (2002 UICC III‐IV	Cisplatin	10:00 h–22:00 h	30	10:00 h–11:00 h	30
					Fluorouracil	22:00 h–10:00 h		11:00 h, 24 h infusion	
Randomized clinical trials (2 × 2)									
Tsuchiya et al. 2016 (Japan)	9	Mean = 51.8	78% vs. 22%	OSCC (III & IV)	Docetaxel	18:30 h–19:30 h	9	10:30 h–11:30 h	9
					Cisplatin	19:30 h–21:30 h		11:30 h–13:30 h	
					Fluorouracil	21:30 h, 24 h infusion		13:30 h, 24 h infusion	
Retrospective cohorts									
Chen et al. 2013 (China)	49	Median = 55	73% vs. 27%	Recurrent and/or metastaticHNSCC (Stage III‐IV))	Paclitaxel	03:00 h–05:00 h	28	Started 09:00 h–11:00 h finished before 17:30 h	21
		Mean = 57.1			Carboplatin	16:00 h–20:00 h		Started 09:00 h–11:00 h finished before 17:30 h	
					Fluorouracil	22:00 h–07:00 h		Started 09:00 h–11:00 h finished before 17:30 h	
Zhang et al. 2021 (China)	150	Mean = 54.60	71% vs. 29%	NPC (2010 UICC III, IVa & IVb)	Cisplatin	10:00 h–22:00 h with peak delivery at 16:00 h	75	Time and duration unspecified	75

Abbreviations: HNSCC, head and neck squamous cell carcinomas; LAHNSCC, locally advanced head and neck squamous cell carcinomas; NPC, nasopharyngeal carcinoma; OSSC, oral squamous cell carcinomas.

#### 3.2.2 Treatment efficacy

Treatment efficacy was significantly better in five of the 13 chrono‐chemotherapy groups^49^, ^50^, ^53^, ^58^, ^60^ (Table 4). Four studies found significant differences in ORR.^49^, ^50^, ^58^, ^60^ Two papers reported significantly higher percentage of complete response (CR) in the chrono‐chemotherapy groups.^49^, ^53^ Six chrono‐chemotherapy groups, including three receiving cisplatin alone did not report an improved treatment efficacy (Table 4, Supplemental Table S7). Both Li et al. (2018) and Tsuchiya et al. (2016) did not investigate treatment efficacy at all.

**TABLE 4:** Chrono‐chemotherapy treatment efficacy and toxicity and adverse events in included studies.

Author/Year	Treatment					Significant differences in treatment efficacy end‐points: intervention versus control; *p* < 0.05	Significant differences in toxicity and adverse events end‐points: intervention versus control; *p* < 0.05
	Drug	Intervention	*n*	Control	*n*		
Taxanes, platinum‐based and antimetabolite chemotherapeutic agents							
Chen et al. 2013 (Retrospective cohort)	Paclitaxel	03:00 h–05:00 h	28	Started 09:00 h–11:00 h finished before 17:30 h	21	• ORR: 71.43% vs. 42.86%	• Leukopenia: Grade I‐II: 28.57% vs. 33.33%; Grade III‐IV: 3.57% vs. 28.57%
	Carboplatin	16:00 h–20:00 h		Started 09:00 h–11:00 h finished before 17:30 h		• OS: 15.3 months vs. 10.6 months	• Neutropenia: Grade I‐II: 21.43% vs. 42.86%
	Fluorouracil	22:00 h–07:00 h		Started 09:00 h–11:00 h finished before 17:30 h			• Stomatitis: Grade III‐IV: 0.0% vs. 23.81%
							• Nausea and vomiting: Grade I‐II: 42.86% vs. 47.62%; Grade III‐IV:0.0% vs. 28.57%
							• Alopecia: Grade I‐II: 3.57% vs. 28.57%
Bi et al. 2015 (RCT 1:1)	Docetaxel	03:30 h–04:30 h	36	Time and duration unspecified	30	PR: 80.6% vs. 50.0%. [induction chemotherapy]	• Leukopenia [Grade I‐IV]: 63.8% vs. 96.6%
	Cisplatin	10:00 h–22:00 h		Time and duration unspecified		CR: 45.5 vs. 20.7%. [concurrent chemoradiotherapy]	• Neutropenia [Grade I‐IV]: 58.3% vs. 83.3%
	Fluorouracil	22:00 h–10:00 h		Time and duration unspecified			• Nausea/Vomiting [Grade I‐IV]: 25% vs. 93.3%
							• Diarrhea [Grade I‐IV]:41.6% vs. 76.6
							• Constipation [Grade I‐IV]: 5.5% vs. 26.6%
							• Oral mucositis [Grade I‐IV]: 2.7% vs. 46.6%
							• Fatigue: Grade I: 83.3% vs. 36.6%; Grade II: 5.5% vs. 43.3%; Grade III: 0% vs. 13%
							• Anorexia: Grade I: 91.6% vs. 36.6%; Grade II: 8.3% vs. 40%; Grade III: 0% vs. 23.3%
							• CD4+/CD8+ ratio – after treatment: 1.58 ± 0.81 vs. 1.15 ± 0.71
Mao et al. 2015 (RCT 1:1)	Docetaxel	06/07:00 h–10:00 h	22	Time and duration unspecified	23	None	• Nausea/Vomiting [Grade ≥ II]: 30.4% vs. 56.5%
	Cisplatin	10:00 h–22:00 h		Time and duration unspecified			• CD4/CD8 count after chemotherapy increased in intervention vs. control
	Fluorouracil	22:00 h–10:00 h		Time and duration unspecified			
Tsuchiya et al. 2016 (RCT −2 × 2)	Docetaxel	18:30 h–19:30 h	9	10:30 h–11:30 h	9	Treatment efficacy was not investigated due to crossover RCT design	Nausea:Grade I: 33.3% vs. 0.0%; Grade II: 33.3% vs. 33.3%; Grade III: 22.2% vs. 66.7%
	Cisplatin	19:30 h–21:30 h		11:30 h–13:30 h			
	Fluorouracil	21:30 h, 24 h infusion		13:30 h, 24 h infusion			
Li et al. 2018 (RCT 1:1)	Docetaxel	18:30 h–19:30 h	16	1 h after breakfast, duration unspecified	16	Treatment efficacy was not investigated	• Neutropenia [Grade IV]: 0.0% vs. ≈50.0%
	Cisplatin	19:30 h −21:30 h		2 h after breakfast, duration unspecified			• Nausea [Grade III]: < 20.0% vs. >60.0%
	Fluorouracil	10:00 h–18:00 h		8 h duration, time unspecified			
Liu et al. 2020 (RCT 1:1)	Docetaxel	03:30 h–04:30 h	66	10:00 h, duration unspecified	69	None	• Hearing loss: Grade I‐II: 22.72% vs. 39.13%
	Cisplatin	10:00 h–22:00 h		10:00 h, duration unspecified			• Dysphagia: Grade I‐II: 0% vs. 8.69%
	Fluorouracil	22:00 h–10:00 h		10:00 h, 24 h infusion			• Neck Fibrosis: Grade I: 4.54% vs. 15.94%
Platinum‐based and antimetabolite chemotherapeutic agents							
Ou‐Yang and Jin 2006 (RCT 1:1)	Cisplatin	10:00 h–22:00 h	30	10:00 h, duration unspecified	30	• Overall CR: 36.7% vs. 20.0%	• Leukopenia (Grade I‐IV): 43.3% vs. 80.0%
	Fluorouracil	22:00 h–10:00 h		10:00 h, duration unspecified		• ORR: 96.0% vs. 69.2%	
Chen et al. 2012 (RCT 1:1)	Oxaliplatin	10:00 h–22:00 h	23	9:00 h, duration unspecified	23	ORR: (95.6% vs. 86.9%)	• Diarrhea [Grade I‐IV]: 13% vs. 34.8%
	Fluorouracil	22:00 h–10:00 h		9:00 h, duration unspecified			• Leukopenia [Grade I‐IV]: 17.4% vs. 34.6%
							• Peripheral neuritis [Grade I‐IV]: 8.6% vs. 26%
Lin et al. 2013 (RCT 1:1)	Cisplatin	10:00 h–22:00 h with peak delivery at 16:00 h	63	10:00 h–22:00 h	61	None	• Anemia: [after first cycle]; Grade I: 17.5% vs. 29.5%; Grade II: 3.2% vs. 9.8%
	Fluorouracil	22:00 h–10:00 h with peak delivery at 04:00 h		22:00 h–10:00 h			• Stomatitis: [after two cycles plus radiotherapy] Grade I: 61.9% vs. 41.0%; Grade II: 0.0% vs. 0.0%; Grade III: 38.1% vs. 59.0%
Gou et al. 2018 (RCT 1:1)	Cisplatin	10:00 h–22:00 h	30	10:00 h– 11:00 h	30	• ORR: 96.7% vs. 73.3%	• Leukocytopenia: Grade I: 30% vs. 43.4%; Grade II: 13.3% vs. 26.7%; Grade III: 0.0% vs. 10.0%
	Fluorouracil	22:00 h–10:00 h		11:00 h, 24 h infusion		• Average local relapse time: 22.6 months vs. 11.9 months	• Thrombocytopenia: Grade I: 23.3% vs. 50.0%; Grade II: 3.3% vs. 6.7%
							• Nausea/vomiting: Grade I: 23.3% vs. 40.0%; Grade II: 16.7% vs. 26.7%
Platinum‐based chemotherapeutic agents							
Verma et al. 2014 (RCT 1:1)	Cisplatin	18:00 h, duration unspecified	30	06:00 h, duration unspecified	30	None	• Nausea/vomiting: Grade III: 6.7% vs. 20.0%.
Zhang et al. 2018 (RCT 1:1)	Cisplatin	10:00 h–22:00 h with peak delivery at 16:00 h	74	10:00 h–14:00 h	74	None	• Nausea: Grade I: 60.9% vs. 43.8%; Grade II: 5.8% vs. 30.1%; Grade 3: 0.0% vs. 2.7%
							• Vomiting: Grade I: 30.4% vs. 17.8%; Grade II: 17.3% vs. 42.5%; Grade III: 0.0% vs. 10.9%
							• Mucositis: Grade I: 30.4% vs. 32.9%; Grade II: 43.5% vs. 39.7%; Grade III: 0.0% vs. 15.1%
							• CD4+/CD8+ ratio (1.65 ± 0.87 vs. 1.17 ± 0.78)
Zhang et al. 2021 (Retrospective Cohort)	Cisplatin	10:00 h–22:00 h with peak delivery at 16:00 h	75	Time and duration unspecified	75	None	• Gastrointestinal reaction: Grade I‐II 70.7% vs. 76.0%; Grade III‐IV: 0,0% vs. 6.7%
							• Oral mucositis: Grade I‐II: 74.6% vs. 81.3%; Grade III‐IV: 1.3% vs. 6.7%
							• CD16 + CD56+ T count (posttreatment): 21.71 ± 9.59 vs. 18.34 ± 10.17

Abbreviations: CR, complete response; ORR, objective response rate; OS, overall survival; PR, partial response.

#### 3.2.3 Toxicity and adverse events

All included studies reported significant reduction of toxicity and adverse events in the chrono‐chemotherapy groups, namely haematological, gastrointestinal, neurological and skin toxicities. Five out of 13 studies reported significant reduction in leukopenia^49^, ^50^, ^53^, ^58^, ^60^ and three out of 13 found significant reduction in neutropenia in chrono‐chemotherapy groups.^53^, ^56^, ^60^ In addition, eight studies out of 13 showed significant reduction in nausea and vomiting severity,^52^, ^53^, ^54^, ^55^, ^56^, ^58^, ^59^, ^60^ and five studies also demonstrated significantly reduced oral mucositis/stomatitis severity in chrono‐chemotherapy groups^51^, ^53^, ^55^, ^60^, ^61^ (Table 4, Supplemental Table S8).

#### 3.2.4 Chrono‐chemotherapy effect on objective response rate

ORR was reported in only eight included chrono‐chemotherapy RCTs. The between‐study heterogeneity variance was estimated at *τ* ^2^ = 0.13 (95% CI: 0.00–2.06), with an *Ι* ^2^ value of 30.5% (95% CI: 0.0%–69.0%) and the prediction interval ranged between 0.17 and 1.77. As shown in Figure 2B, HNC patients who underwent chrono‐chemotherapy had 44% less overall risk of lower ORR than those who had nontime‐stipulated chemotherapy (RR: 0.56, 95% CI: 0.28–1.14, *p* > 0.05). This effect estimate was insignificant with inconclusive confidence intervals. However, subgroup analysis revealed HNC patients who underwent chrono‐chemotherapy using platinum‐based and antimetabolite agents had 73% less risk of lower ORR than those who had nontime‐stipulated chemotherapy (RR: 0.27, 95% CI: 0.09–0.84, *p* < 0.05).

#### 3.2.5 Chrono‐chemotherapy effect on toxicity and adverse events (Grade ≥3)

Nine RCTs were analyzed for toxicity and adverse events. The estimated variance of between‐study heterogeneity was *τ* ^2^ = 0 (95% CI: 0.00–0.25), with an *Ι* ^2^ value of 0% (95% CI: 0.0%–40.2%), and the prediction interval ranged between 0.46 and 0.75. HNC patients who underwent chrono‐chemotherapy had 41% less risk of overall toxicity and adverse events than those who had nontime‐stipulated chemotherapy (RR: 0.59, 95% CI: 0.47–0.72, *p* < 0.05). As shown in Figures S1 and S2, subgroup analysis showed that chrono‐chemotherapy had 32% and 60% less risk of haematological and gastrointestinal toxicities and adverse events than nontime‐stipulated chemotherapy, respectively (RR: 0.68, 95% CI: 0.55–0.83, *p* < 0.05 and RR: 0.40, 95% CI: 0.25–0.63, *p* < 0.05).

## 4 DISCUSSION

This systematic review included 14 randomized clinical trials, two nonrandomized clinical trials and six retrospective studies. Approximately 59% (13 of 22) of included studies investigated chrono‐chemotherapy while 41% (9 of 22) assessed chrono‐radiotherapy in HNC patients. Overall, included studies were heterogenous in terms of study design, intervention (dose, regimen and duration) and population. Most chrono‐radiotherapy studies evaluating toxicity and adverse events (six [75%] out of eight) reported significant reduction in adverse events, namely severe oral mucositis, in chrono‐radiotherapy (morning) groups compared to evening radiotherapy. However, none of these studies reported improved treatment efficacy. On the other hand, about one‐third (five [38.5%] of 13) of chrono‐chemotherapy studies reported significantly improved treatment efficacy, while all studies showed significant reduction in toxicity and adverse events in chrono‐chemotherapy groups compared to nontime‐stipulated chemotherapy. Our systematic review's findings are mainly in accordance with four published reviews (two with meta‐analysis) investigating chrono‐chemotherapy (*n* = 3)^26^, ^27^, ^29^ and chrono‐radiotherapy (*n* = 1)^28^ in different cancers, including HNC. Shuboni‐Mulligan et al. (2019) included three RCTs and six retrospective studies in their systematic review and concluded that chrono‐radiotherapy may reduce treatment symptoms (adverse events) in highly proliferative tissues.^28^ They also found that chrono‐radiotherapy was inconsistently beneficial for treatment efficacy outcomes.^28^ In Shuboni‐Mulligan et al.'s systematic review, treatment efficacy was similar among both groups. The second review by Printezi et al. (2022) included 14 RCTs and reported that chronomodulated chemotherapy could reduce toxicities while maintaining efficacy.^26^ In addition, they found two studies that had inconsistent effect on toxicities and one other study showed that standard chemotherapy had fewer side effects than chronomodulated chemotherapy. However, the included chrono‐chemotherapy studies in our systematic review demonstrated consistent reduction of toxicity and adverse events across all chrono‐chemotherapy groups.

Our meta‐analyses included RCTs only (Chrono‐radiotherapy = 3 and Chrono‐chemotherapy = 9). Chrono‐radiotherapy had significantly lower risk of developing severe oral mucositis, while chrono‐chemotherapy using platinum‐based and antimetabolite agents (*n* = 4) had significantly lower risk of stable or progressive cancer after treatment. Also, toxicities such as anaemia, diarrhoea, leukopenia, nausea and vomiting and neutropenia were analyzed in this study. We found that chrono‐chemotherapy, regardless of chemotherapeutic regimen, had significantly lower risk of toxicity and adverse events (*n* = 9). These results are partially aligned with the third systematic review and meta‐analysis investigating the effect of chrono‐chemotherapy on ORR and overall toxicities in colorectal cancer in seven RCTs.^29^ Similar to our study, the authors also reported significant differences between chrono‐chemotherapy and nonchrono‐chemotherapy groups in terms of haematological toxicities.^29^ Another systematic review and meta‐analysis by Huang et al. (2017) included six RCTs that reported decreased risk of neutropenia and mucositis favouring chrono‐chemotherapy in patients with metastatic colorectal cancer.^27^

HNC patients who underwent chrono‐radiotherapy had 31% less risk of developing severe oral mucositis (grade ≥3) compared to evening radiotherapy. This is due to normal cells in the morning (i.e., G_1_ phase) being more resistant to radiation‐induced adverse events. On the other hand, treatment efficacy showed no particular advantage to chrono‐radiotherapy. The maintained treatment efficacy is likely attributed to the unpredictable nature of cancer cells (including their circadian rhythms) as compared to healthy cells.^19^ These observations could explain our findings alongside those of other studies, which revealed that chrono‐radiotherapy (morning) significantly reduces the severity of oral mucositis compared to evening radiotherapy, while maintaining similar treatment efficacy.^28^, ^30^

Chrono‐chemotherapy had 73% and 41% less risk of reduced ORR and increased toxicity and adverse events, respectively. Compared to preclinical studies, findings from human trials are more challenging to ascertain whether the superior treatment efficacy was achieved directly by circadian variation of chemotherapeutic targets or indirectly by the increased dosage due to reduced toxicity.^26^ However, all included chrono‐chemotherapy in this review had similar doses between their experimental groups, and ORR was significantly improved in five studies,^49^, ^50^, ^53^, ^58^, ^60^ while six studies reported maintained treatment efficacy among groups.^51^, ^52^, ^54^, ^55^, ^57^, ^61^ Nevertheless, more preclinical studies are still needed to investigate the circadian variation of chemotherapeutic targets. Also, future clinical trials could increase dose intensities utilizing chrono‐chemotherapy, thus indirectly improving treatment efficacy.

This systematic review had several limitations. First, there were notable differences among included studies in terms of study design, intervention (dose, regimen and duration) and population. Moreover, there was no research group that investigated both chrono‐radiotherapy and chrono‐chemotherapy, so we separated our results and analyses for each intervention respectively. Second, due to scarcity of evidence and number of studies published in the literature, we did not exclude non‐RCTs and retrospective cohorts. However, we critically appraised the quality of all included studies and it ranged from fair (13 out of 22) to good (9 out of 22). Third, publication bias could not be assessed due to the small sample size of included studies, and our meta‐analysis was comprised of a small number of studies as well. However, several precautions were taken in our statistical models to ensure that the pooled effect sizes are not overestimated. Nonetheless, the prediction intervals revealed promising results for future studies. Finally, most chrono‐chemotherapy studies (84.6%) investigated Chinese patients, while 55% of chrono‐radiotherapy were performed on Indian patients. Therefore, multicentric and multi‐ethnic clinical studies are still needed to support the use of chrono‐radiotherapy and chrono‐chemotherapy in HNC.

While evidence from this systematic review and meta‐analysis is promising, the studies' methodological limitations warrant caution in interpreting the results. First, most included studies had major flaws in their study design such as the lack of blinded treatment assignment, blinded outcome assessment and treatment groups allocation concealment. Although blinding participants to treatment assignment in a timely intervention and a placebo‐controlled study design might prove to be challenging in terms of feasibility, the other abovementioned design flows could significantly increase the risk bias, thus compromising the internal validity of these papers. However, it is feasible to have investigators that are blinded to treatment allocation, assignment and outcome assessment. Second, HNC treatment protocols often combine chemotherapy with radiotherapy and patients rarely get prescribed one treatment modality without the other. For example, two thirds of patients in studies investigating chrono‐radiotherapy received chemotherapy too and all patients in studies evaluating chrono‐chemotherapy had also received radiotherapy. Indeed, none of the included studies investigated both chrono‐radiotherapy and chrono‐chemotherapy nor did the studies investigating chrono‐radiotherapy consider the administration time of chemotherapy and vice versa. In addition, all patients in chrono‐chemotherapy studies had multiple cycles of nontime‐stipulated chemotherapy. Such overlap between treatment modalities and protocols will influence treatment efficacy and adverse events outcomes, thus possibly diluting effect size of interventions. For instance, Kuriakose et al. (2016) found that a patient who had radiotherapy with concurrent chemotherapy reported significantly higher severity of oral mucositis as opposed to radiotherapy alone.^45^ Thus, future studies should incorporate optimum administration time for both chemo and radiotherapy in all treatment cycles.

Moreover, circadian rhythm modifiers such as age and sex are necessary to consider when conducting chronotherapy studies. It is well documented that there are differences in the circadian clock between sexes and among age groups, and they play an important role in the effect observed with chronotherapy.^28^, ^62^ For instance, Bjarnason et al. (2009) (included in this review) reported that optimal timing for chrono‐radiotherapy to reduce oral mucositis might be sex/gender‐specific.^40^ Such differences could be due to sex/gender‐specific genes involved in different pathways like the cell cycles.^63^ Further, Giacchetti et al. (2012) showed that chrono‐chemotherapy in colorectal cancer was superior in males compared to females.^64^ More recently, Cui et al. (2023) reported that in patients with nasopharyngeal carcinoma, chemotherapy related adverse events were much worse in women while their overall prognosis was better than men.^65^ In addition, females diagnosed with different cancers had higher incidence of toxicities and adverse events from platinum‐based chemotherapeutic agents, which could warrant dose adjustments based on sex/gender.^66^ These differences could influence the reported findings since most patients of included studies were males and older in age. Therefore, researchers should implement circadian‐based protocols in future studies to improve the reliability and internal validity.

Furthermore, treatment protocols greatly differed between included papers. In chrono‐radiotherapy studies, inter and intra irradiation duration and administration time varied between papers. Also, chrono‐chemotherapy studies had dissimilar number of chemotherapeutics used, duration and administration time. These contrasting intervention properties directly affect both treatment efficacy and toxicity and adverse events. High doses of the intervention, for instance, might increase treatment efficacy but could worsen the side effects. In this systematic review, we found significant effect on objective response rate favouring chrono‐chemotherapy only in studies that prescribed platinum‐based and antimetabolite chemotherapeutic agents (RR: 0.27, 95% CI: 0.09–0.84, *p* < 0.05). Nonetheless, such variations in treatment protocols are to be expected as different subtypes of head and neck cancers and stages necessitate combined treatment modalities. However, subgroup analyses for various HNC stages and doses, although homogenously distributed across groups, were mainly absent in most included studies. Subgroup analyses are important to accurately estimate the effect of chronotherapy on desired outcomes and pooled outcome measures could subsequently dilute the effect. For example, Bjarnason et al. (2009) observed significant reduction in adverse events only in a subgroup of patients receiving ≥66 Gy of radiation.^40^ Further, Verma et al. (2014) reported different complete tumour (T1‐T4) and node (N1‐N3) responses, some of which demonstrated superior effect favouring chrono‐chemotherapy and others were comparable or worse.^52^ In addition, Ou‐Yang and Jin (2006) achieved 100% ORR in nasopharyngeal cancer stage I and II.^49^ However, in stage III and IV, 96% and 69.2% ORR was reported for chrono‐chemotherapy and control groups, respectively.^49^ Such results are to be expected as advanced cancers tend to be harder to cure when compared to early stages, and delays in diagnosis and treatment have been associated with higher mortality, thus exhibiting worse treatment efficacy and adverse events.^67^ In addition, it is well‐known that HPV status strongly influences survival rates in both chemotherapy and radiotherapy, and HPV‐positive tumours generally are more responsive to therapy.^7^ However, the included studies did not consider HPV status as a variable in their analysis. Therefore, optimized study arms, design and inclusion and exclusion criteria with a robust statistical plan should be present in future clinical trials.

### 4.1 Recommendations for future chronotherapy trial designs

1‐ Optimized study arms, design and inclusion and exclusion criteria with a robust statistical plan should be present.
2‐ Circadian‐based protocols should be implemented to improve the reliability and internal validity.
3‐ Optimum administration time for both chemotherapy and radiotherapy in all treatment cycles should be incorporated.
4‐ Future clinical trials could increase dose intensities utilizing chrono‐chemotherapy, thus indirectly improving treatment efficacy.
5‐ Multicentric and multi‐ethnic clinical studies are still needed to support the use of chrono‐radiotherapy and chrono‐chemotherapy in HNC.

## 5 CONCLUSION

In conclusion, only chrono‐chemotherapy studies show evidence of improved treatment efficacy, while in chrono‐radiotherapy it was maintained. Both chrono‐radiotherapy and chrono‐chemotherapy studies provide evidence of reduced toxicity and adverse events. However, optimized multicentric blinded randomized clinical trials for both chrono‐radiotherapy and chrono‐chemotherapy are needed to confirm these findings. Finally, optimized study arms, design and inclusion and exclusion criteria with a robust statistical plan should be present in future clinical trials.

## AUTHOR CONTRIBUTIONS

**Mohammad Abusamak:** Conceptualization; methodology; writing – original draft; writing – review and editing; formal analysis; data curation; validation; visualization; project administration; software; investigation. **Abdel‐Azez Abu‐Samak:** Data curation; project administration; writing – review and editing. **Wenji Cai:** Data curation; writing – review and editing. **Haider Al‐Waeli:** Formal analysis; writing – review and editing; validation. **Faez Saleh Al‐Hamed:** Formal analysis; validation. **Mohammad Al‐Tamimi:** Data curation. **Malik Juweid:** Formal analysis; writing – review and editing. **Akhilanand Chaurasia:** Writing – review and editing. **Belinda Nicolau:** Investigation; supervision; writing – review and editing. **Faleh Tamimi:** Investigation; supervision; writing – review and editing.

## CONFLICT OF INTEREST STATEMENT

The authors declare no conflict of interest.

## Supporting information


**Data S1.** Supplementary Information.

## Data Availability

The data and R script are available upon reasonable request from the corresponding authors.

## References

[1] Bray F , Laversanne M , Sung H , et al. Global cancer statistics 2022: GLOBOCAN estimates of incidence and mortality worldwide for 36 cancers in 185 countries. CA Cancer J Clin. 2024;74(3):229‐263. doi:10.3322/caac.21834 38572751

[2] Ferlay J , Colombet M , Soerjomataram I , et al. Estimating the global cancer incidence and mortality in 2018: GLOBOCAN sources and methods. Int J Cancer. 2019;144(8):1941‐1953. doi:10.1002/ijc.31937 30350310

[3] Mody MD , Rocco JW , Yom SS , Haddad RI , Saba NF . Head and neck cancer. Lancet. 2021;398(10318):2289‐2299. doi:10.1016/S0140-6736(21)01550-6 34562395

[4] Chow Laura QM . Head and neck cancer. N Engl J Med. 2020;382(1):60‐72. doi:10.1056/NEJMra1715715 31893516

[5] Pfister DG , Spencer S , Adelstein D , et al. Head and neck cancers, version 2.2020, NCCN clinical practice guidelines in oncology. J Natl Compr Cancer Netw. 2020;18(7):873‐898. doi:10.6004/jnccn.2020.0031 32634781

[6] Cohen N , Fedewa S , Chen AY . Epidemiology and demographics of the head and neck cancer population. Oral Maxillofac Surg Clin. 2018;30(4):381‐395. doi:10.1016/j.coms.2018.06.001 30078696

[7] Tabatabaeian H , Bai Y , Huang R , Chaurasia A , Darido C . Navigating therapeutic strategies: HPV classification in head and neck cancer. Br J Cancer. 2024;131(2):220‐230. doi:10.1038/s41416-024-02655-1 38643337 PMC11263586

[8] Warnakulasuriya S , Greenspan JS , eds. Textbook of Oral Cancer: Prevention, Diagnosis and Management. Springer International Publishing; 2020. doi:10.1007/978-3-030-32316-5

[9] Osazuwa‐Peters N , Simpson MC , Zhao L , et al. Suicide risk among cancer survivors: head and neck versus other cancers. Cancer. 2018;124(20):4072‐4079. doi:10.1002/cncr.31675 30335190

[10] Givens DJ , Karnell LH , Gupta AK , et al. Adverse events associated with concurrent Chemoradiation therapy in patients with head and neck cancer. Arch Otolaryngol Neck Surg. 2009;135(12):1209‐1217. doi:10.1001/archoto.2009.174 20026818

[11] Franco P , Potenza I , Schena M , et al. Induction chemotherapy and sequential concomitant chemo‐radiation in locally advanced head and neck cancers: how induction‐phase intensity and treatment breaks May impact on clinical outcomes. Anticancer Res. 2015;35(11):6247‐6254. 26504058

[12] Kubicek GJ , Machtay M . New advances in high‐technology radiotherapy for head and neck cancer. Hematol Oncol Clin North Am. 2008;22(6):1165‐1180. doi:10.1016/j.hoc.2008.08.014 19010266

[13] Lee N , Chuang C , Quivey JM , et al. Skin toxicity due to intensity‐modulated radiotherapy for head‐and‐neck carcinoma. Int J Radiat Oncol. 2002;53(3):630‐637. doi:10.1016/S0360-3016(02)02756-6 12062606

[14] Tan M , Chen Y , Du T , et al. Assessing the impact of charged particle radiation therapy for head and neck adenoid cystic carcinoma: a systematic review and meta‐analysis. Technol Cancer Res Treat. 2024;23:15330338241246653. doi:10.1177/15330338241246653 38773763 PMC11113043

[15] Manavi MA , Fathian Nasab MH , Mohammad Jafari R , Dehpour AR . Mechanisms underlying dose‐limiting toxicities of conventional chemotherapeutic agents. J Chemother. 2024;1‐31. doi:10.1080/1120009X.2023.2300217 38179685

[16] Farshadi E , van der Horst GTJ , Chaves I . Molecular links between the circadian clock and the cell cycle. J Mol Biol. 2020;432(12):3515‐3524. doi:10.1016/j.jmb.2020.04.003 32304699

[17] Bjarnason GA , Jordan RCK , Wood PA , et al. Circadian expression of clock genes in human Oral mucosa and skin: association with specific cell‐cycle phases. Am J Pathol. 2001;158(5):1793‐1801. doi:10.1016/S0002-9440(10)64135-1 11337377 PMC1891949

[18] Bjarnason GA , Jordan RCK , Sothern RB . Circadian variation in the expression of cell‐cycle proteins in human Oral epithelium. Am J Pathol. 1999;154(2):613‐622. doi:10.1016/S0002-9440(10)65306-0 10027418 PMC1849996

[19] Fu L , Kettner NM . Chapter nine‐the circadian clock in cancer development and therapy. In: Gillette MU , ed. Progress in Molecular Biology and Translational Science. Vol 119. Biological Timing in Health and Disease. Academic Press; 2013:221‐282. doi:10.1016/B978-0-12-396971-2.00009-9 23899600 PMC4103166

[20] Amiama‐Roig A , Verdugo‐Sivianes EM , Carnero A , Blanco JR . Chronotherapy: circadian rhythms and their influence in cancer therapy. Cancer. 2022;14(20):5071. doi:10.3390/cancers14205071 PMC9599830 36291855

[21] Lin Y , Wang S , Zhou Z , Guo L , Yu F , Wu B . Bmal1 regulates circadian expression of cytochrome P450 3a11 and drug metabolism in mice. Commun Biol. 2019;2(1):378. doi:10.1038/s42003-019-0607-z 31633069 PMC6795895

[22] Lévi F , Focan C , Karaboué A , et al. Implications of circadian clocks for the rhythmic delivery of cancer therapeutics. Adv Drug Deliv Rev. 2007;59(9):1015‐1035. doi:10.1016/j.addr.2006.11.001 17692427

[23] Lévi F , Okyar A , Dulong S , Innominato PF , Clairambault J . Circadian timing in cancer treatments. Annu Rev Pharmacol Toxicol. 2010;50:377‐421. doi:10.1146/annurev.pharmtox.48.113006.094626 20055686

[24] Jacobs BAW , Deenen MJ , Pluim D , et al. Pronounced between‐subject and circadian variability in thymidylate synthase and dihydropyrimidine dehydrogenase enzyme activity in human volunteers. Br J Clin Pharmacol. 2016;82(3):706‐716. doi:10.1111/bcp.13007 27161955 PMC5338101

[25] Granda TG , Filipski E , D'Attino RM , et al. Experimental chronotherapy of mouse mammary adenocarcinoma MA13/C with docetaxel and doxorubicin as single agents and in Combination1. Cancer Res. 2001;61(5):1996‐2001. 11280758

[26] Printezi MI , Kilgallen AB , Bond MJG , et al. Toxicity and efficacy of chronomodulated chemotherapy: a systematic review. Lancet Oncol. 2022;23(3):e129‐e143. doi:10.1016/S1470-2045(21)00639-2 35240088

[27] Huang Y , Yu Q , Liu Y , et al. Efficacy and safety of chronomodulated chemotherapy for patients with metastatic colorectal cancer: a systematic review and meta‐analysis. Asia Pac J Clin Oncol. 2017;13(2):e171‐e178. doi:10.1111/ajco.12456 26892158

[28] Shuboni‐Mulligan DD , Breton G , Smart D , Gilbert M , Armstrong TS . Radiation chronotherapy—clinical impact of treatment time‐of‐day: a systematic review. J Neurooncol. 2019;145(3):415‐427. doi:10.1007/s11060-019-03332-7 31729636 PMC8130840

[29] Nassar A , Abdelhamid A , Ramsay G , Bekheit M . Chronomodulated Administration of Chemotherapy in advanced colorectal cancer: a systematic review and meta‐analysis. Cureus. 2023;15(3):e36522. doi:10.7759/cureus.36522 37090313 PMC10120847

[30] Abusamak M , Al‐Tamimi M , Al‐Waeli H , et al. Chronotherapy in dentistry: a scoping review. Chronobiol Int. 2023;40(5):684‐697. doi:10.1080/07420528.2023.2200495 37052061

[31] Page MJ , McKenzie JE , Bossuyt PM , et al. The PRISMA 2020 statement: an updated guideline for reporting systematic reviews. BMJ. 2021;372:n71. doi:10.1136/bmj.n71 33782057 PMC8005924

[32] Lockwood C , Munn Z , Porritt K . Qualitative research synthesis: methodological guidance for systematic reviewers utilizing meta‐aggregation. Int J Evid Based Healthc. 2015;13(3):179‐187. doi:10.1097/XEB.0000000000000062 26262565

[33] Mantel N , Haenszel W . Statistical aspects of the analysis of data from retrospective studies of disease. J Natl Cancer Inst. 1959;22(4):719‐748. doi:10.1093/jnci/22.4.719 13655060

[34] Paule RC , Mandel J . Consensus values and weighting factors. J Res Natl Bur Stand. 1982;87(5):377‐385. doi:10.6028/jres.087.022 PMC6768160 34566088

[35] Knapp G , Hartung J . Improved tests for a random effects meta‐regression with a single covariate. Stat Med. 2003;22(17):2693‐2710. doi:10.1002/sim.1482 12939780

[36] Higgins JPT , Thompson SG . Quantifying heterogeneity in a meta‐analysis. Stat Med. 2002;21(11):1539‐1558. doi:10.1002/sim.1186 12111919

[37] Cochran WG . Some methods for strengthening the common χ2 tests. Biometrics. 1954;10(4):417‐451. doi:10.2307/3001616

[38] Balduzzi S , Rücker G , Schwarzer G . How to perform a meta‐analysis with R: a practical tutorial. BMJ Ment Health. 2019;22(4):153‐160. doi:10.1136/ebmental-2019-300117 PMC10231495 31563865

[39] Viechtbauer W . Conducting meta‐analyses in *R* with the **metafor** package. J Stat Softw. 2010;36(3):1‐48. doi:10.18637/jss.v036.i03

[40] Bjarnason GA , MacKenzie RG , Nabid A , et al. Comparison of toxicity associated with early morning versus late afternoon radiotherapy in patients with head‐and‐neck cancer: a prospective randomized trial of the National Cancer Institute of Canada clinical trials group (HN3). Int J Radiat Oncol. 2009;73(1):166‐172. doi:10.1016/j.ijrobp.2008.07.009 18805649

[41] Goyal M , Shukla P , Gupta D , et al. Oral mucositis in morning vs. evening irradiated patients: a randomised prospective study. Int J Radiat Biol. 2009;85(6):504‐509. doi:10.1080/09553000902883802 19412843

[42] Lavanya L , Arulponni T . Correlation of oral mucositis with timing of radiation in head and neck cancer‐a prospective randomized study on chronoradiotherapy. J Radiat Cancer Res. 2021;12(1):10‐14. doi:10.4103/jrcr.jrcr_62_20

[43] Elzahi MSE , Attia SE , Elazab SH . Timing of daily radiotherapy for cases of head and neck cancer: does it make difference? J Cancer Tumor Int. 2020;10(1):12‐16. doi:10.9734/jcti/2020/v10i130118

[44] Ponna DR , Sobita Devi Y , Baidya K , et al. Assessing the different patterns of radiation reactions in head and neckcancer patients undergoing radiation therapy at different times of the day‐in the morning versus evening. Int J Adv Res. 2021;9(7):284‐291. doi:10.21474/IJAR01/13127

[45] Kuriakose DVG , Anand DAS , Jayakumar DK , Radhakrishnan DA , Meloot DSS . Influence of circadian rhythm in radiation induced Mucositis in head and neck malignancies. IOSR J Pharm. 2016;6(11):21‐26.

[46] Gu F , Farrugia MK , Duncan WD , et al. Daily time of radiation treatment is associated with subsequent Oral Mucositis severity during radiotherapy in head and neck cancer patients. Cancer Epidemiol Biomarkers Prev. 2020;29(5):949‐955. doi:10.1158/1055-9965.EPI-19-0961 32098893 PMC7898770

[47] Brolese EK , Cihoric N , Bojaxhiu B , et al. The impact of delivery daytime and seasonality of radiotherapy for head and neck cancer on toxicity burden. Radiother Oncol. 2021;158:162‐166. doi:10.1016/j.radonc.2021.02.039 33667582

[48] Elicin O , Koller Brolese E , Bojaxhiu B , et al. The prognostic impact of daytime and seasonality of radiotherapy on head and neck cancer. Radiother Oncol. 2021;158:293‐299. doi:10.1016/j.radonc.2021.04.004 33848563

[49] Ou‐Yang J , Jin F . A pilot study of chronochemotherapy for nasopharyngeal carcinoma. Chin J Clin Oncol. 2006;3(6):423‐427. doi:10.1007/s11805-006-0132-y

[50] Chen H , Jin F , Wu W , et al. Single‐center randomized controled phase II trial of combined chronomodulated chemotherapy and radiotherapy for nasopharyngeal carcinoma. Chin J Clin Oncol. 2012;39(6):336‐339. doi:10.3969/j.issn.1000-8179.2012.06.010

[51] Lin HX , Hua YJ , Chen QY , et al. Randomized study of sinusoidal chronomodulated versus flat intermittent induction chemotherapy with cisplatin and 5‐fluorouracil followed by traditional radiotherapy for locoregionally advanced nasopharyngeal carcinoma. Chin J Cancer. 2013;32(9):502‐511. doi:10.5732/cjc.013.10004 23816561 PMC3845560

[52] Verma Y , Chauhan AK , Singh H , Sabharwal R , Bharti M , Kaur P . Chronomodulated chemotherapy and concomitant radiotherapy, for the management of locally advanced, head and neck squamous cell carcinoma. International Journal of Pharmacy & Life Sciences. 2014;5(3):1015‐1022.

[53] Bi T , Jin F , Wu W , et al. Phase II clinical trial of two different modes of administration of the induction chemotherapy for locally advanced nasopharyngeal carcinoma. Zhonghua Zhong Liu Za Zhi. 2015;37(9):676‐681. 26813432

[54] Mao Z , Jin F , Wu W , et al. Clinical study of chrono‐chemotherapy in treating nasopharyngeal carcinoma patients with distant metastasis at preliminary diagnosis. Chin J Clin Oncol. 2015;24:709‐715.

[55] Zhang PX , Jin F , Li ZL , et al. A randomized phase II trial of induction chemotherapy followed by cisplatin chronotherapy versus constant rate delivery combined with radiotherapy. Chronobiol Int. 2018;35(2):240‐248. doi:10.1080/07420528.2017.1397684 29215933

[56] Li Y , Yu H , Ke Y , et al. Effect of administration time on toxicity induced by DCF in patients with Oral squamous cell carcinoma. Anti‐Tumor Pharm. 2018;8(1):44‐48.

[57] Liu KQ , Jin F , Jiang H , et al. Analysis of follow‐up results of chrono‐chemotherapy or conventional chemotherapy combined with intensity modulated radiotherapy in locally advanced nasopharyngeal carcinoma. Zhonghua Zhong Liu Za Zhi. 2020;42(2):133‐138. doi:10.3760/cma.j.issn.0253-3766.2020.02.009 32135648

[58] Gou XX , Jin F , Wu WL , et al. Induction chronomodulated chemotherapy plus radiotherapy for nasopharyngeal carcinoma: a phase II prospective randomized study. J Cancer Res Ther. 2018;14(7):1613‐1619. doi:10.4103/jcrt.JCRT_883_17 30589048

[59] Tsuchiya Y , Ushijima K , Noguchi T , et al. Influence of a dosing‐time on toxicities induced by docetaxel, cisplatin and 5‐fluorouracil in patients with oral squamous cell carcinoma; a cross‐over pilot study. Chronobiol Int. 2018;35(2):289‐294. doi:10.1080/07420528.2017.1392551 29144178

[60] Chen D , Cheng J , Ma Y , Yang K , Yang F . Retrospective analysis of chronomodulated chemotherapy versus conventional chemotherapy with paclitaxel, carboplatin, and 5‐fluorouracil in patients with recurrent and/or metastatic head and neck squamous cell carcinoma. OncoTargets Ther. 2013;6:1507‐1514. doi:10.2147/OTT.S53098 PMC3810446 24187501

[61] Zhang S , Teng T , Liao G , et al. Efficacy of induction chemotherapy combined with chrono‐chemotherapy and intensity‐modulated radiotherapy on locally advanced nasopharyngeal carcinoma. J BUON. 2021;26(3):774‐780. 34268935

[62] Bailey M , Silver R . Sex differences in circadian timing systems: implications for disease. Front Neuroendocrinol. 2014;35(1):111‐139. doi:10.1016/j.yfrne.2013.11.003 24287074 PMC4041593

[63] Bjarnason GA , Seth A , Wang Z , Blanas N , Straume M , Martino TA . Diurnal rhythms (DR) in gene expression in human oral mucosa: implications for gender differences in toxicity, response and survival and optimal timing of targeted therapy (Rx). J Clin Oncol. 2007;25(18_suppl):2507. doi:10.1200/jco.2007.25.18_suppl.2507

[64] Giacchetti S , Dugué PA , Innominato PF , et al. Sex moderates circadian chemotherapy effects on survival of patients with metastatic colorectal cancer: a meta‐analysis. Ann Oncol. 2012;23(12):3110‐3116. doi:10.1093/annonc/mds148 22745214

[65] Cui L , Chen Z , Zeng F , et al. Impact of sex on treatment‐related adverse effects and prognosis in nasopharyngeal carcinoma. BMC Cancer. 2023;23(1):1146. doi:10.1186/s12885-023-11564-0 38007428 PMC10676584

[66] Marcu LG . Gender and sex‐related differences in Normal tissue effects induced by platinum compounds. Pharmaceuticals. 2022;15(2):255. doi:10.3390/ph15020255 35215367 PMC8876358

[67] Hanna TP , King WD , Thibodeau S , et al. Mortality due to cancer treatment delay: systematic review and meta‐analysis. BMJ. 2020;371:m4087. doi:10.1136/bmj.m4087 33148535 PMC7610021

