# Supplementary material for: Chronotherapy in head and neck cancer: A systematic review and meta‐analysis
Source: Int J Cancer. 2024 Nov 7;156(5):1015–32. doi: 10.1002/ijc.35234 (PMC11701392; doi:10.1002/ijc.35234)
Supplement: Supplementary file 1 — Data S1. Supplementary Information. [file IJC-156-1015-s001.pdf]

Chronotherapy in Head and Neck Cancer: A Systematic Review and Meta-Analysis

Mohammad Abusamak, Abdel-Azez Abu-Samak, Wenji Cai, Haider Al-Waeli, Faez Saleh Al-Hamed, Mohammad Al-Tamimi, Malik Juweid, Akhilanand Chaurasia, Belinda Nicolau, Faleh Tamimi.

Supplementary Information

Table of contents

Supplementary materials & methods
Supplemental Table 1. Search strategy for each database…………………………………………………….. 2-4
Supplementary tables
Supplemental Table 2. Quality critical appraisal of included studies………………………….…………… 5&6
Supplemental Table 3. Demographics and characteristics of radiotherapy included studies........ 7
Supplemental Table 4. Chrono-radiotherapy treatment response in included studies…….…... 8&9
Supplemental Table 5. Chrono-radiotherapy toxicity and adverse events in included studies……….......... 10-12
Supplemental Table 6. Chemotherapeutic regimen included chemotherapy studies……............ 13
Supplemental Table 7. Chrono-chemotherapy treatment response in included studies…..….. 14-16
Supplemental Table 8. Chrono-chemotherapy toxicity in included studies……………..……..…… 17-20
Supplementary figures
Supplemental Figure 1. Effect of chrono-chemotherapy on gastrointestinal toxicity and adverse events…….. 21
Supplemental Figure 2. Effect of chrono-chemotherapy on gastrointestinal toxicity and adverse events……..…… 22

Supplemental Table 1. Search strategy for each database.

MEDLINE(Ovid)

Steps

1. exp Circadian Rhythm/
2. exp Circadian Clocks/
3. exp Biological Clocks/
4. exp Chronotherapy/
5. ((circadian or biological or diurnal or nyctohemeral or twenty-four hour or 24-hour or ultradian) adj1 (rhythm? or clock?)).tw,kf.
6. chronotherapy.tw,kf.
7. (morning? or afternoon? or evening?).tw,kf.
8. (time? of day or morning? or afternoon? or evening?).tw,kf.
9. or/1-8
10. exp Dentists/ or exp Dentistry/ or exp Dental Clinics/ or exp Dental Auxiliaries/
11. (dentist* or denturist* or ((dental or oral) adj3 (health or care or surgeon? or office? or clinic? or assistant? or nurse? or hygien* or practitioner? or professional? or auxiliar*))).tw,kf.
12. exp Dental Health Services/
13. exp Stomatognathic Diseases/
14. exp Stomatognathic System/
15. exp Oral Health/
16. (((periodontal or periapical or stomatognathic or oral or mouth or dental) adj3 disease?) or periodontitis or pericoronitis or peri-implantitis or caries or ((tooth or teeth) adj3 (loss or mobility)) or (gingival adj3 (h?emorrhage? or overgrowth or recession)) or gingivitis or (oral adj1 (hygiene or health))).tw,kf.
17. exp "Head and Neck Neoplasms"/ or exp Facial Neoplasms/ or exp Mouth Neoplasms/ or exp Otorhinolaryngologic Neoplasms/ or exp Tracheal Neoplasms/
18. ((cancer* or tumo?r* or neoplas* or metaplas* or carcinoma* or metastasi* or squamous cell carinoma? or SCC or HNSCC or malignan*) adj5 (head or neck or uadt or "upper aero-digestive" or "upper aerodigestive" or face or facial or oral* or intra-oral* or intraoral* or mouth or buccal or gingiv* or gum* or lip? or labial* or palat* or lingual* or mandib* or maxill* or jaw? or tongue* or glossal* or otor?inolaryngolog* or throat or ear? or auricle* or auricular or larynx* or laryngeal* or nose* or nasal* or paranasal* or sinus or hypopharynx or hypopharyngeal* or nasopharynx or nasopharyngeal* or oropharynx or oropharyngeal or tonsil* or trachea* or cheek* or pharynx or pharyngeal or retromolar or alveolar or tonsil* or sinonasal or sinus* or vestib* or piriform or post-cricoid or glottic or subglottic or superglottic or transglottic or "unknown primary" or trigone or maxillofacial*)).tw,kf.
19. (exp Head/ or exp Neck/) and exp Neoplasms/
20. or/10-19
21. 9 and 20

EMBASE

Steps

1. exp biological rhythm/
2. exp chronotherapy/
3. ((circadian or biological or diurnal or nyctohemeral or twenty-four hour or 24-hour or ultradian) adj1 (rhythm? or clock?)).tw,kf.
4. chronotherapy.tw,kf.
5. (morning? or afternoon? or evening?).tw,kf.
6. (time? of day or morning? or afternoon? or evening?).tw,kf.
7. or/1-6
8. exp dentist/ or exp dentistry/ or exp dental clinic/ or exp dental auxiliary/
9. (dentist* or denturist* or ((dental or oral) adj3 (health or care or surgeon? or office? or clinic? or assistant? or nurse? or hygien* or practitioner? or professional? or auxiliar*))).tw,kf.

10\. exp dental clinic/

12\. exp mouth disease/

12\. exp stomatognathic system/

13\. (((periodontal or periapical or stomatognathic or oral or mouth or dental) adj3 disease?) or periodontitis or pericoronitis or peri-implantitis or caries or ((tooth or teeth) adj3 (loss or mobility)) or (gingival adj3 (h?emorrhage? or overgrowth or recession)) or gingivitis or (oral adj1 (hygiene or health))).tw,kf.

14\. exp "head and neck cancer"/

15. ((cancer* or tumo?r* or neoplas* or metaplas* or carcinoma* or adenoma* or adenocarcinoma* or metastasi* or sarcoma* or ameloblastoma* or (odontogenic* adj3 keratocyst*) or malignan*) adj5 (head or neck or face or facial or oral* or intra-oral* or intraoral* or "intra oral*" or mouth or buccal or gingiv* or gum* or lip or lips or labial* or palat* or palatal* or salivary or parotid or lingual* or sublingual* or sub-lingual* or mandib* or submandib* or sub-mandib* or maxill* or jaw or jaws or tongue* or glossal* or nose* or nasal* or paranasal* or sinus or nasopharynx or nasopharyngeal* or tonsil* or cheek*)).tw.
16. (exp head/ or exp neck/) and (exp cancer/ or exp tumor/)
17. or/8-16
18. 7 and 17

CINAHL
Steps
S1. (MH "Circadian Rhythm+")
S2. (MH "Biological Clocks+")
S3. (MH "Chronotherapy+")
S4. TI ((circadian or biological or diurnal or nyctohemeral or twenty-four hour or 24-hour or ultradian) N1 (rhythm# or clock#)) OR AB ((circadian or biological or diurnal or nyctohemeral or twenty-four hour or 24-hour or ultradian) N1 (rhythm# or clock#))
S5. TI (time# of day OR morning# or afternoon# or evening# or chronotherapy) OR AB (time# of day OR morning# or afternoon# or evening# or chronotherapy)
S6. S1 OR S2 OR S3 OR S4 OR S5
S7. (MH "Dentists+") or (MH "Dentistry+") or (MH "Dental Clinics+") or (MH "Dental Auxiliaries+")
S8. TI (dentist* or denturist* or ((dental or oral) N3 (health or care or surgeon# or office# or clinic# or assistant# or nurse# or hygien* or practitioner# or professional# or auxiliar*))) OR AB (dentist* or denturist* or ((dental or oral) N3 (health or care or surgeon# or office# or clinic# or assistant# or nurse# or hygien* or practitioner# or professional# or auxiliar*)))
S9. (MH "Dental Health Services+")
S10. (MH "Stomatognathic Diseases+")
S11. (MH "Oral Health+")
S12. (((periodontal or periapical or stomatognathic or oral or mouth or dental) N3 disease#) or periodontitis or pericoronitis or peri-implantitis or caries or ((tooth or teeth) N3 (loss or mobility)) or (gingival N3 (h#emorrhage# or overgrowth or recession)) or gingivitis or (oral N1 (hygiene or health))) OR AB (((periodontal or periapical or stomatognathic or oral or mouth or dental) N3 disease#) or periodontitis or pericoronitis or peri-implantitis or caries or ((tooth or teeth) N3 (loss or mobility)) or (gingival N3 (h#emorrhage# or overgrowth or recession)) or gingivitis or (oral N1 (hygiene or health)))
S13. (MH "Head and Neck Neoplasms+")
S14. TI ((cancer* or tumo#r* or neoplas* or metaplas* or carcinoma* or metastasi* or squamous cell carinoma# or SCC or HNSCC or malignan*) N5 (head or neck or uadt or "upper aero-digestive" or "upper aerodigestive" or face or facial or oral* or intra-oral* or intraoral* or mouth or buccal or gingiv* or gum* or lip# or labial* or palat* or lingual* or mandib* or maxill* or jaw# or tongue* or glossal* or otor#inolaryngolog* or throat or ear# or auricle* or auricular or larynx* or laryngeal* or nose* or nasal* or paranasal* or sinus or hypopharynx or hypopharyngeal* or nasopharynx or nasopharyngeal* or oropharynx or oropharyngeal or tonsil* or trachea* or cheek* or pharynx or pharyngeal or retromolar or alveolar or tonsil* or sinonasal or sinus* or vestib* or piriform or post-cricoid or glottic or subglottic or superglottic or transglottic or "unknown primary" or trigone or maxillofacial*)) OR AB ((cancer* or tumo#r* or neoplas* or metaplas* or carcinoma* or metastasi* or squamous cell carinoma# or SCC or HNSCC or malignan*) N5 (head or neck or uadt or "upper aero-digestive" or "upper aerodigestive" or face or facial or oral* or intra-oral* or intraoral* or mouth or buccal or gingiv* or gum* or lip# or labial* or palat* or lingual* or mandib* or maxill* or jaw# or tongue* or glossal* or otor#inolaryngolog* or throat or ear# or auricle* or auricular or larynx* or laryngeal* or nose* or nasal* or paranasal* or sinus or hypopharynx or hypopharyngeal* or nasopharynx or nasopharyngeal* or oropharynx or oropharyngeal or tonsil* or trachea* or cheek* or pharynx or pharyngeal or retromolar or alveolar or tonsil* or sinonasal or sinus* or vestib* or piriform or post-cricoid or glottic or subglottic or superglottic or transglottic or "unknown primary" or trigone or maxillofacial*))
S15. S7 OR S8 OR S9 OR S10 OR S11 OR S12 OR S13 OR S14
S16. S6 AND S15

SCOPUS
(INDEXTERMS (circadian OR biological AND clock OR chronotherapy) OR TITLE-ABS-KEY-AUTH ((circadian OR biological OR diurnal OR nyctohemeral OR "twenty-four hour" OR 24-hour OR ultradian) W/1 (rhythm* OR clock*)) OR TITLE-ABS-KEY-AUTH (chronotherapy OR "time* of day" OR morning* OR afternoon* OR evening*)) AND ((INDEXTERMS (dentist* OR dental or "head and neck neoplasms" or stomatognathic or "oral health") OR TITLE-ABS-KEY-

AUTH (dentist* OR denturist* OR ((dental OR oral) W/3 (health OR care OR surgeon* OR office* OR clinic* OR assistant* OR nurse? OR hygien* OR practitioner* OR professional* OR auxiliar*)))) OR (TITLE-ABS-KEY-AUTH (((periodontal OR periapical OR stomatognathic OR oral OR mouth OR dental) W/3 disease*) OR periodontitis OR pericoronitis OR peri-implantitis OR caries OR ((tooth OR teeth) W/3 (loss OR mobility)) OR (gingival W/3 (hemorrhage* OR haemorrhage* OR overgrowth OR recession)) OR gingivitis OR (oral W/1 (hygiene OR health)))) OR (TITLE-ABS-KEY-AUTH(cancer* or tumor* or tumour* or neoplas* or metaplas* or carcinoma* or metastasi* or "squamous cell carinoma*"or SCC or HNSCC or malignan*) W/5 (head or neck or uadt or "upper aero-digestive" or "upper aerodigestive" or face or facial or oral* or intra-oral* or intraoral* or mouth or buccal or gingiv* or gum* or lip or lips or labial* or palat* or lingual* or mandib* or maxill* or jaw or jaws or tongue* or glossal* or otorinolaryngolog* or otorhinolaryngolog* or throat or ear or ears or auricle* or auricular or larynx* or laryngeal* or nose* or nasal* or paranasal* or sinus or hypopharynx or hypopharyngeal* or nasopharynx or nasopharyngeal* or oropharynx or oropharyngeal or tonsil* or trachea* or cheek* or pharynx or pharyngeal or retromolar or alveolar or tonsil* or sinonasal or sinus* or vestib* or piriform or post-cricoid or glottic or subglottic or superglottic or transglottic or "unknown primary" or trigone or maxillofacial*)) OR (TITLE-ABS-KEY-AUTH (intra-oral* OR intraoral* OR buccal OR gingiv* OR gum OR gums OR palat* OR lingual* OR mandib* OR maxill* OR glossal* OR otorinolaryngolog* OR otorhinolaryngolog OR larynx* OR laryngeal* OR sinus OR hypopharynx OR hypopharyngeal* OR nasopharynx OR nasopharyngeal* OR oropharynx OR oropharyngeal OR tonsil* OR trachea* OR cheek* OR pharynx OR pharyngeal OR retromolar OR alveolar OR tonsil* OR sinonasal OR sinus* OR vestib* OR piriform OR post-cricoid OR glottic OR subglottic OR superglottic OR transglottic OR "unknown primary" OR trigone OR maxillofacial*))) AND NOT DBCOLL(medl)

Supplemental Table 2. Quality critical appraisal of included studies

First Author/ Year<br>**RCT**	Q1	Q2	Q3	Q4	Q5	Q6	Q7	Q8	Q9	Q10	Q11	Q12	Q13	Overall Quality
Chrono-chemotherapy														
Verma et al. 2014	Low	High	Low	High	High	High	Low	Low	High	Low	Low	Unclear	Low	Fair
Zhang et al. 2018	Low	Low	Low	High	High	High	Low	Low	High	Low	Low	Low	Low	Fair
Lin et al. 2013	Low	High	Low	High	High	High	Low	Low	High	Low	Low	Low	Low	Fair
Tsuchiya et al. 2018	Low	High	Low	High	High	High	Low	Low	High	Low	Low	Low	Low	Fair
Chen et al. 2012	Low	High	Low	High	High	Low	Low	Low	High	Low	Low	Low	Low	Fair
Bi et al. 2015	Low	Low	Low	High	High	Low	Low	Low	High	Low	Low	Low	Low	Good
Gou et al. 2018	Low	High	Low	High	High	High	Low	Low	High	Low	Low	Low	Low	Fair
Li et al. 2018	Low	High	Low	High	High	Low	Low	Low	High	Low	Low	Low	Low	Fair
Liu et al. 2020	Low	High	Low	High	High	Low	Low	Low	High	Low	Low	Low	Low	Fair
Mao et al. 2015	Low	High	Low	High	High	Low	Low	Low	High	Low	Low	Low	Low	Fair
Ou-Yang et al 2006	Low	Low	Low	High	High	High	Low	Low	High	Low	Low	Low	Low	Fair
Chrono-radiotherapy														
Goyal et al. 2009	Low	Low	Low	High	High	Low	Low	Low	Low	Low	High	Low	Low	Good
Bjarnason et al. 2009	Low	Low	Low	High	High	Low	High	Low	Low	Low	High	Low	Low	Fair
Lavanya et al. 2021	Low	Low	Low	High	High	Low	High	Low	High	Low	High	Unclear	Low	Fair

**Non – RCT**	Q1	Q2	Q3	Q4	Q5	Q6	Q7	Q8	Q9	Overall Quality
Chrono-radiotherapy										
Elzahi et al. 2020	Low	Low	Low	Low	Low	Low	Low	Low	Low	Good
Ponna et al. 2021	Low	Low	Low	Low	Low	Low	Low	Low	Low	Good

Low Risk (green)
Unclear Risk (yellow)
High Risk (red)

**Cohorts**	Q1	Q2	Q3	Q4	Q5	Q6	Q7	Q8	Q9	Q10	Q11	Overall Quality
Chrono-chemotherapy												
Chen D et al. 2013	Low	Low	Low	High	High	Low	Low	Low	Low	Unclear	Low	Good
Zhang S et al. 2021	Low	Low	Low	High	High	Low	Low	Low	Low	Unclear	Low	Good
Chrono-radiotherapy												
Gu et al. 2020	Low	Low	Low	Low	Low	Low	Low	Low	Low	Unclear	Low	Good
Kuriakose et al. 2016	Low	Low	Low	High	High	Unclear	Low	High	High	High	Low	Fair
Brolese et al. 2021	Low	Low	Low	Low	Low	Unclear	Low	Low	Low	Low	Low	Good
Elicin et al. 2021	Low	Low	Low	High	High	Low	Low	Low	Low	Unclear	Low	Good

Abbreviations: RCT: Randomized Controlled Trials; Q: Question tool. See supplemental document, pages 6, for assessment questions.

Joanna Briggs Institute risk of bias assessment questions for RCT:

Q1. Was true randomization used for assignment of participants to treatment groups?
Q2. Was allocation to treatment groups concealed?
Q3. Were treatment groups similar at the baseline?
Q4. Were participants blind to treatment assignment?
Q5. Were those delivering treatment blind to treatment assignment?
Q6. Were outcomes assessors blind to treatment assignment?
Q7. Were treatment groups treated identically other than the intervention of interest?
Q8. Was follow up complete and if not, were differences between groups in terms of their follow up adequately described and analyzed?
Q9. Were participants analyzed in the groups to which they were randomized?
Q10. Were outcomes measured in the same way for treatment groups?
Q11. Were outcomes measured in a reliable way?
Q12. Was appropriate statistical analysis used?
Q13. Was the trial design appropriate, and any deviations from the standard RCT design (individual randomization, parallel groups) accounted for in the conduct and analysis of the trial?

Joanna Briggs Institute risk of bias assessment questions for non-RCT:

Q1. Is it clear in the study what is the 'cause' and what is the 'effect' (i.e. there is no confusion about which variable comes first)?
Q2. Were the participants included in any comparisons similar?
Q3. Were the participants included in any comparisons receiving similar treatment/care, other than the exposure or intervention of interest?
Q4. Was there a control group?
Q5. Were there multiple measurements of the outcome both pre and post the intervention/exposure?
Q6. Was follow up complete and if not, were differences between groups in terms of their follow up adequately described and analyzed?
Q7. Were the outcomes of participants included in any comparisons measured in the same way?
Q8. Were outcomes measured in a reliable way?
Q9. Was appropriate statistical analysis used?

Joanna Briggs Institute risk of bias assessment questions for Cohorts:

Q1. Were the two groups similar and recruited from the same population?
Q2. Were the exposures measured similarly to assign people to both exposed and unexposed groups?
Q3. Was the exposure measured in a valid and reliable way?
Q4. Were confounding factors identified?
Q5. Were strategies to deal with confounding factors stated?
Q6. Were the groups/participants free of the outcome at the start of the study (or at the moment of exposure)?
Q7. Were the outcomes measured in a valid and reliable way?
Q8. Was the follow up time reported and sufficient to be long enough for outcomes to occur?
Q9. Was follow up complete, and if not, were the reasons to loss to follow up described and explored?
Q10. Were strategies to address incomplete follow up utilized?
Q11. Was appropriate statistical analysis used?

Supplemental Table 3 Demographics and characteristics of radiotherapy included studies.

Author/Year	Tumor	Irradiation Dose and Technique
Bjarnason et al. 2009	HNSSC only	1) 50 Gy in 25 fractions<br>2) 60 Gy in 25 or 30 fractions<br>3) 66 Gy in 33 fractions<br>4) 70 Gy in 35 fractions<br>RT technique: unspecified
Goyal et al. 2009	HNSCC (92%)<br>Others (8%)	≥ 60 Gy<br>RT technique: Cobalt using parallel and opposite two fields
Elzahi et al. 2020	Unspecified	65-70 Gy<br>RT technique: unspecified
Lavanya et al. 2021	Unspecified	66 Gy in 30 fractions<br>RT technique: IMRT
Ponna et al. 2021	HNSSC (63%)<br>Others (37%)	66-70 Gy in 30 -35 fractions<br>RT technique: Cobalt-60 using EBRT
Kuriakose et al. 2016	HNSSC only	60-66 Gy in 30-33 fractions.<br>RT technique: Lateral parallel pair/unilateral field and a low anterior neck field
Gu et al. 2020	HNSSC only	1) 70 Gy in 35 fractions for primary tumor.<br>2) 56 Gy to the elective nodes in 35 fractions.<br>RT technique: IMRT
Brolese et al. 2021	HNSSC only	≥ 60 Gy in 2 Gy daily fraction<br>RT technique<br>3D conformal (47%) Static-field IMRT (38.2%)<br>VMAT (57.1%)
Elicin et al. 2021	HNSSC only	≥ 60 Gy in 2 Gy daily fraction<br>RT technique<br>3D conformal (9.2%) Static-field IMRT (36.2%)<br>VMAT (54.7%)

Abbreviations: HNSSC, Head and Neck Squamous Cell Carcinoma; RT, Radiotherapy; IMRT, Intensity-modulated radiation therapy; EBRT, External beam radiation therapy; VMAT, Volumetric modulated arc therapy.

Supplemental Table 4. Chrono-radiotherapy treatment response in included studies.

Author/Year	Treatment		Treatment Response Endpoints (Grading Scale)	Insignificant differences (MRT vs ERT; p> 0.05)
	Morning RT	Evening RT		
Bjarnason et al. 2009	08:00h – 10:00h (n= 104)	16:00h – 18:00h (n= 101)	1) Duration of locoregional control<br>2) Survival rate	1) 2-year locoregional control: 64% vs 60%<br>2) 2-year survival rate: 61.1% vs 64.1%
Goyal et al. 2009	08:00h – 11:00h (n= 88)	15:00h – 18:00h (n= 89)	CR, PR, SD & PD (WHO criteria)	CR (51% vs 46%)<br>PR (48% vs 53%)<br>SD (1% vs 1%)
Ponna et al. 2021	08:00h – 10:00h (n= 62)	15:00h – 17:00h (n= 62)	1- CR, PR, SD & PD at 1 months and 6 months (RECIST criteria)<br>2- Overall 2-year survival	1) 1 month:<br>CR: 79% vs 69%<br>PR: 11.3% vs 12.9%<br>SD & PD: Unclear<br>2) 6 months:<br>- CR, PR, SD & PD: Unclear<br>- Disease Free Survival: 83% vs 79%<br>- Survival with Disease: 4.8% vs 8%<br>- Overall Survival: 88.6% vs 87%<br>3) 2-year Overall Survival: 69.4% vs 53.2%
Elicin et al. 2021	00:00h – 12:00h (n= 354)	12:00h – 00:00h (n= 301)	1) Loco-regional control<br>2) Progression-free survival<br>3) Overall survival	Hazard ratio (AM/PM):<br>1) Loco-regional control [1.09 (0.81–1.48)]<br>2) Progression-free survival [1.11 (0.88–

	1.39)]<br>3) Overall survival [1.24 (0.96–1.62)]

Abbreviations: RT, radiotherapy; MRT, Morning Radiotherapy; ERT, Afternoon/Evening Radiotherapy; WHO, World Health Organization; CR, Complete Response; PR, Partial Response; SD, Stable Disease; PD, Progressive Disease; RECIST, Response Evaluation Criteria in Solid Tumours. Note: There were no significant differences between groups.

Supplemental Table 5. Chrono-radiotherapy toxicity and adverse events in included studies

Author/Year	Treatment		Toxicity Endpoints (Grading Scale)	Significant differences (MRT vs ERT; p<0.05)	Insignificant differences (MRT vs ERT; p>0.05)	Symptomatic Tx
	Morning RT	Evening RT				
Bjarnason et al. 2009	08:00h – 10:00h (n= 104)	16:00h – 18:00h (n= 101)	1) Incidence of Grade III or greater oral mucositis (RTOG)<br>2) Interval to the development of Grade II or greater mucositis<br>3) Duration of various grades of oral mucositis<br>4) Proportion of patients with ≥ 1 Tx days lost because of toxicity<br>5) Incidence of other acute and late toxicities	1) Incidence of Grade III or greater oral mucositis:<br>- Subgroup received ≥ 66 Gy of radiation (n=111): 44.6%: vs 67.3%<br>- Patients who smoked during therapy (n=53): 42.9% vs 76%<br>2) Interval to the development of Grade II or greater mucositis:<br>- Subgroup received ≥ 66 Gy of radiation (n=111): >7.9 weeks vs 5.6 weeks (median)	1) Incidence of Grade III or greater oral mucositis: 52.9% vs 62.4%<br>2) Median interval to mucositis development: (weeks)<br>- Grade II or greater: 2.4 vs 2.6<br>- Grade III or greater: 7 vs 5.6<br>3) Median duration of mucositis (weeks):<br>- Grade II: 2.1 vs 2<br>- Grade III: 2.9 vs 2.1<br>- Grade IV: 4.9 vs 2.1<br>4) ≥ 1 Tx days lost because of toxicity: 3% vs 7.1%<br>5) Incidence of other Grade 3 or greater acute toxicity<br>- Dysphagia during Tx: 8.6% vs 16.7%	NSAIDs. Xylocaine gel or Xylocaine viscous was allowed for painful oral ulcerations
Goyal et al. 2009	08:00h – 11:00h (n= 88)	15:00h – 18:00h (n= 89)	1) Incidence of oral mucositis grade III & IV. (RTOG)<br>2) Progression rate of oral mucositis - 7 weeks<br>3) Incidence of skin reaction (RTOG)	Progression rate of oral mucositis: ERT > MRT at week 4 and 7.	1) Incidence of mucositis grade III and IV (26% vs 38%)<br>2) Incidence of skin reaction grade III and IV (10% vs 14%)	Oral anaesthetic gels and analgesics
Elzahi et al. 2020	06:00h – 08:00h (n= 80)	13:00h – 15:00h (n= 80)	Soreness Quality Score (SQS) for mouth and throat soreness severity	1) SQS 3 & 4 (34% vs 64%)<br>2) Overall median SQS (2 vs 3)	None	Unspecified
Lavanya et al. 2021	08:00h – 11:00h (n= 32)	17:00h – 20:00h (n= 32)	1) Incidence of oral mucositis (RTOG)<br>2) Weight loss	None	1) 2nd week:<br>Grade I: 40.6% vs 31.2%<br>Grade II: 0% vs 3.10%<br>2) 5th week<br>Grade III: 0% vs 6.20%<br>3) 6th week<br>Grade III: 21.90% vs 25%<br>4) First Follow up (2 weeks after Tx)<br>Grade III: 25% vs 34.4%	Gentian violet, benzocaine gel, sucralfate gargle, and oral analgesics along with serratiopeptidase

					5) 2nd Follow up (6 weeks after Tx)<br>Grade II: 84.4% vs 90.6%<br>6) Mean weight loss (3.91kg vs 4.25kg)	
Ponna et al. 2021	08:00h – 10:00h (n= 62)	15:00h – 17:00h (n= 62)	Onset, duration, and severity of oral mucositis (RTOG)	1) Onset:<br>-Week 2: 51.6% vs 83.9%<br>-Week 3: 67.7% vs 90.3%<br>-Week 7: 88.7% vs 98.4%<br>2) Severity of mucositis (end of Tx)<br>-Grade III: 25.8% vs 46.8%<br>-Grade IV: 1.6% vs 12.9%<br>3) Median time to develop grade III or IV oral mucositis:<br>4.7 weeks vs 5.8 weeks	1) Onset:<br>-Week 4: 85.5% vs 91.9%<br>-Week 5: 90.3% vs 96.8%<br>-Week 6: 91.9% vs 96.8%<br>2) Severity of mucositis (end of Tx)<br>-Grade 0: 8.1%% vs 1.6%<br>-Grade I: 19.4% vs 6.5%<br>-Grade II: 45.2% vs 32.3%	Unspecified
Kuriakose et al. 2016	08:00h – 11:00h (n= 73)	17:00h – 20:00h (n= 69)	1) Incidence of oral mucositis grade III or IV. (RTOG)<br>2) RT interruptions due to toxicity<br>3) Mean time to develop oral mucositis grade III or IV.	1) Incidence of oral mucositis grade III & IV: 42.47% vs 60.9%<br>2) RT interruptions due to toxicity: 17.8% vs 42%	Mean time to develop oral mucositis grade III or IV: 6 weeks vs 5 weeks	Unspecified
Gu et al. 2020	08:30h –09:30h (n= 32)<br>09:30h–10:30h (n=32)<br>10:30h–11:30h (n= 36)<br>11:30h –12:30h (n=26)	12:30h–14:00h (n=25)<br>14:00h–15:00h (n=25)<br>15:00h–16:30h (n=14)	1) Severity of mouth throat soreness - MTS (OMDQ)	1) Average treatment timing and repeated MTS measures: [LSmeans ±SD]<br>- 08:30h to < 09:30h [1.33±0.11] vs 14:00h to < 15:00h [1.83±0.11]<br>2) Maximum MTS [LSmeans ±SE]:<br>- Lowest maximum MTS: 08:00h to <09:30h [2.24±0.15] vs highest maximum MTS: 12:30h to <14:00h [2.71±0.17]<br>3) Incidence of severe oral mucositis (3+ MTS %):<br>- 08:30h –<09:30h: 50%<br>- 09:30h–<10:30h: 62.5%<br>- 10:30h–<11:30h: 66.7%<br>- 11:30h –<12:30h: 61.5%<br>- 12:30h–<14:00h: 68%<br>- 14:00h–<15:00h: 72%<br>- 15:00h–<16:30h: 57.1%	None	Baking soda/salt oral rinses, diphenhydramine lidocaine antacid solution. NSAIDs, acetaminophen, and narcotics.

Brolese et al. 2021	00:00h – 12:00h (n= 336)	12:00h – 00:00h (n= 281)	Acute and late toxicity scores (CTCAE)	Mean acute toxicity scores (AM vs PM): 1.71 vs 1.88	Mean late toxicity scores (AM vs PM): 0.31 vs 0.41	Unspecified

Abbreviations: RT, Radiotherapy; MRT, Morning Radiotherapy; ERT, Afternoon/Evening Radiotherapy; RTOG, Radiation Therapy Oncology Group; NSAIDs, Nonsteroidal anti-inflammatory drugs; Tx, Treatment; CTCAE, Common Terminology Criteria for Adverse Events.

Supplemental Table 6. Chemotherapeutic regimen included chemotherapy studies.

Author/Year	Chemotherapeutic Regimen
Lin et al. 2013	12-hour continuous infusion of Cisplatin (20mg/m^2) or 5-Fu (750mg/m^2) for five days, repeated every three weeks for two 21-days cycles.
Verma et al. 2014	Flat infusion of Cisplatin (30mg/m^2) weekly. Duration of intervention and no. of cycles was not reported.
Zhang et al. 2018	- Two 21-days cycles of induction chemotherapy [Day 1: Docetaxel (75 mg/m^2 and cisplatin 75mg/m^2; Day 1-5: 5-Fu 750mg/m^2 constant infusion – 120h)].<br>- Two to three 21-days cycles of concurrent chemotherapy [Day 1: Cisplatin 100mg/m^2].
Tsuchiya et al. 2016	Two cycles of continuous infusion; Day 1: Docetaxel (60mg/m2; 1h infusion) and Cisplatin (60mg/m2; 2h infusion); Day 1-5: 5- Fu (600mg/m2; 24h infusion)
Gou et al. 2018	Two 14-days cycles of induction chemotherapy: Day 1-3: Cisplatin 80mg/m^2, 5-fu 1000mg/m^2 plus Citrovorum Factor (CF) 200mg/m^2
Zhang et al. 2021	- Two 21-days cycles of induction chemotherapy [Day 1: Docetaxel (75 mg/m^2 and Cisplatin 75mg/m^2; Day 1-5: 5-Fu 750mg/m^2 constant infusion – 120h)].<br>- Two to three 21-days cycles of concurrent chemotherapy [Day 1: Cisplatin 100mg/m^2].
Chen et al. 2013	Each course 3- 4 weeks: Day 1: Paclitaxel 150 mg/m2 and Carboplatin 350 mg/m2; Day 1-5: 5- Fu 800 mg/m2. (up to 6 times)
Ou-Yang et al. 2006	Two 14-days cycles of induction chemotherapy: Day 1: Cisplatin 80mg/m^2; Day1-3: 5-fu 750 mg/m^2 plus Citrovorum Factor (CF) 200mg/m^2
Mao et al. 2015	- Two 21-28 days cycles of induction chemotherapy [Day 1: Docetaxel (75 mg/m^2; Day 1-5: Cisplatin 75mg/m^2; Day 1-5: 5-Fu 750mg/m^2– 120h)].<br>- Two 21-days cycles of concurrent chemotherapy [Day 1-2: Cisplatin 100mg/m^2].<br>- After one month of concurrent chemotherapy, two 21-28 days cycles of adjunct chemotherapy [Day 1: Docetaxel (75 mg/m^2; Day 1-5: Cisplatin 75mg/m^2; Day 1-5: 5-Fu 750mg/m^2 infusion – 120h)].
Li et al. 2018	- One 21-days cycle of induction chemotherapy [Day 1: Docetaxel infusion (60mg/m2, 1h) and Cisplatin infusion (60 mg/m2, 2h]; Day 1-5: 5-Fu (600 mg/m2, 8h)
Chen et al. 2012	Daily dose of 130mg/m^3 of oxaliplatin from 10:00 to 22:00 for three days, 1000mg/m^2.d of 5-fluorouracil. At least two cycles (one cycle= 14days) treatment.
Bi et al. 2015	TPF regimen with 2 cycles of induction chemotherapy in a 21-28 days/cycle. [Day 1: Docetaxel 75mg/m^2; Day 1-5: Cisplatin 75mg/m^2, 5-Fu 750mg/m^2.d]
Liu et al. 2020	Two cycles of induced chemotherapy (including Docetaxel, Cisplatin, and 5-Fu every 21 days). Docetaxel 75mg/m^2, Cisplatin 75mg/m^2, 5-Fu 750mg/m^2 (5 days)

Abbreviations: 5-Fu, Fluorouracil; CF, Citrovorum Factor.

Supplemental Table 7. Chrono-chemotherapy treatment response in included studies.

Author/Year		Treatment	Treatment Response Endpoints (Grading Scale)	Insignificant differences ≥ 10 % (Intervention vs Control; p ≥0.05)
Lin et al. 2013 (RCT 1:1)	I	Sinusoidal chronomodulated infusion; Cisplatin (10:00h – 22:00h with peak delivery at 16:00h) & 5-Fu (22:00h – 10:00h with peak delivery at 04:00h) [n= 63]	CR, PR, SD, PD, RR, OS, PFS and DMFS (WHO criteria)	None
	C	Intermittent constant rate infusion; 5-Fu (10:00h – 22:00h) & Cisplatin (22:00h – 10:00h) [n=61]		
Verma et al. 2014 (RCT 1:1)	I	Flat infusion of Cisplatin at 18:00h [n=30]	CR, PR, Overall disease complete response [Complete node response + Complete tumor response] (Criteria unspecified)	- Complete tumour response rate (40.0% vs 26.7%)<br>- Overall disease complete response (26.7% vs 13.3%)
	C	Flat infusion of Cisplatin at 06:00h [n=30]		
Zhang et al. 2018 (RCT 1:1)	I	Sinusoidal chronomodulated infusion; Cisplatin (10:00h – 22:00h with peak delivery at 16:00h) [n= 69]	CR, DMFS, RR, OS, PR, PD, PFS, SD (RECIST 1.1)	None
	C	flat infusion of Cisplatin (10:00h – 14:00h) [n= 73]		
Gou et al. 2018 (RCT 1:1)	I	Day 1-3: Infusion of Cisplatin (10:00h-22:00h); 5-Fu (22:00h – 10:00h) [n=30]	CR, PR, SD, PD, OS, LRFS, DMFS. (WHO criteria)	- Nasopharyngeal control rate: 6-year 53.3% vs 43.3%.<br>- Nodal control rate: 1-year 93.3% vs 83.3%; 3-year; 73.3% vs 63.3%.<br>- Average metastasis time: 12.4 months vs 15.5 months.<br>- OS: 5-year: 53.3% vs 43.3%; 10-year: 43.4% vs 33.3%<br>- LRFS: 5-year: 80% vs 70%
	C	Day 1-3: Infusion of Cisplatin (10:00h- 11:00h); 5-Fu (11:00h, 24h infusion) [n=30]		

Zhang et al. 2021 (Retro)	I	Sinusoidal chronomodulated infusion; Cisplatin (10:00h – 22:00h with peak delivery at 16:00h) [n=75]	CR, PR, SD, PD, ORR. (WHO criteria)	None
	C	Conventional intravenous instilling of Cisplatin (Time and duration were not reported) [n=75]		
Chen et al. 2013 (Retro)	I	Day 1: Paclitaxel infusion 03:00h – 05:00h and Carboplatin infusion 16:00h – 2-:00h. Day 1-5: 5-Fu continuous infusion 22:00h to 07:00h. [n=28]	CR, PR, SD, PD, ORR, OS and PFS. (WHO criteria)	PFS: 11.6 months vs 7.2 months
	C	Day 1: Paclitaxel, Carboplatin. Day 1-5: 5-Fu. Started 09:00h – 11:00h and completed before 17:30h. [n=21]		
Ou-Yang et al. 2006 (RCT 1:1)	I	Day 1: Infusion of Cisplatin (10:00h-22:00h); Day 1-3: 5-Fu (22:00h – 10:00h) [n=30]	CR, PR, SD & PD. (WHO criteria 2002)	CR:<br>- Stage I/II: 80.0% vs 50.0%<br>- Stage III/IV: 28.0% vs 15.4%
	C	Day 1: Infusion of Cisplatin (10:00h, duration unspecified); Day 1-3: 5-Fu (10:00h –22:00h); [n=30]		
Mao et al. 2015 (RCT 1:1)	I	Day 1: Docetaxel infusion before Cisplatin for 3-4h duration; Day 1-5: Cisplatin continuous infusion (10:00h – 22:00h); Day 1-5: 5-Fu (22:00h – 10:00h) [n=22]	CR, PR, SD & PD. (2000 RECIST criteria)	- PR: 45.5% vs 60.9%<br>- SD: 50.0% vs 39.1%<br>- CR+PR: 50.0% vs 60.9%
	C	Day 1: Docetaxel infusion before Cisplatin for 3-4h duration; Day 1: Cisplatin continuous infusion *(*duration unspecified); Day 1-5: 5-		

		Fu (24h continuous infusion) [n=23]		
Chen et al, 2012 (RCT 1:1)	I	Day 1-3: Oxaliplatin (10:00h-22:00h) and 5-Fu (22:00h-10:00h) [n=23]	CR, PR, SD & PD. (WHO criteria 1981)	None
	C	Day 1-3: Oxaliplatin and 5-Fu start at 9:00h per day [n=23]		
Bi et al. 2015 (RCT 1:1)	I	Day 1: Docetaxel (03: 30h-04: 30h); Day 1-5: Cisplatin (10:00h-22:00h); 5-Fu (22:00h-10:00h) [36]	CR, PR, SD & PD. (2000 RECIST criteria)	Before concurrent chemoradiotherapy:<br>- SD: 19.4 vs 50%.
	C	Day 1: Docetaxel and Cisplatin infusion; Day 1-5: 5-Fu continuous infusion (120h in total) [30]		After concurrent chemoradiotherapy:<br>- PR: 51.5% vs 75.9%.
Liu et al. 2020 (RCT 1:1)	I	Day 1: Docetaxel (03:30h-04:30h); Day 1-5: Cisplatin (10:00h-22:00h); 5-Fu (22:00h-10:00h) [66]	OS, PFS, LRFS, and DMFS. (1-year & 3-year) [Criteria unspecified]	None
	C	Day 1: Docetaxel and Cisplatin Day 1-5: 5-Fu (120h in total) [69]		

Abbreviations: 5-Fu, Fluorouracil; WHO, World Health Organization; RECIST, Response Evaluation Criteria in Solid Tumours; CR, Complete Response; PR, Partial Response; SD, Stable Disease; PD, Progressive Disease; ORR, Overall Response Rate; OS, Overall Survival; PFS, Progression Free Survival; LRFS, Locoregional Relapse-Free Survival; DMFS, Distant Metastasis-Free Survival

Supplemental Table 8. Chrono-chemotherapy toxicity in included studies

Author/Year		Treatment	Toxicity Endpoints (Grading Scale)	Insignificant differences ≥ 10 % (Intervention vs Control; p≥0.05)
Lin et al. 2013	I	Sinusoidal chronomodulated infusion; Cisplatin (10:00h – 22:00h with peak delivery at 16:00h) & 5-Fu (22:00h – 10:00h with peak delivery at 04:00h) [n= 63]	Hematologic toxicity [Anemia, Leucopenia, Neutropenia and Thrombocytopenia], Renal toxicity [Serum Creatinine], Hepatic toxicity [ALT elevation and AST elevation], Gastrointestinal toxicity [Anorexia, Diarrhea, Vomiting and Stomatitis], Weight loss and Radiodermatitis (NCIC CTC version 3.0)	None
	C	Intermittent constant rate infusion; 5-Fu (10:00h – 22:00h) & Cisplatin (22:00h – 10:00h) [n=61]		
Verma et al. 2014	I	Flat infusion of Cisplatin at 18:00h [n=30]	Anemia, Leukopenia, Mucositis, Weight loss, Nausea/vomiting, Radiodermatitis and Overall severe toxicity [Grade III & IV] (RTOG)	- ≥ 10% Weight loss: 3.3% vs 13.3%<br>- Late [6 months f/u] skin reactions: ≈60.0% vs 80.0%)
	C	Flat infusion of Cisplatin at 06:00h [n=30]		
Zhang et al. 2018	I	Sinusoidal chronomodulated infusion; Cisplatin (10:00h – 22:00h with peak delivery at 16:00h) [n= 69]	Anemia, Leukopenia, Neutropenia, Thrombocytopenia, ALT elevation, AST elevation, Hyperbilirubinemia, Serum Creatinine, Nausea, Vomiting, Mucositis, Xerostomia, Radiodermatitis, Dysphagia, Laryngeal edema, Weight loss, CD3+ count, CD4+ count, CD4+/CD8+ ratio, CD8+ count, CD16+CD56+ count, and CD19+ count (CTCAE version 3.0)	- Anaemia: Grade I: 47.8% vs 37.0%; Grade II: 14.5% vs 30.1%.<br>- Xerostomia: Grade I: 37.7% vs 47.9%; Grade II: 2.9% vs 30.9%.<br>- Dysphagia: Grade I: 43.5% vs 27.4%; grade II: 0% vs 11.0%.
	C	flat infusion of Cisplatin (10:00h – 14:00h) [n= 73]		
Tsuchiya et al. 2016	I	Day 1: Docetaxel infusion (18:30h – 19:30h) and Cisplatin (19:30h-21:30h); Day 1-5: 5- Fu (Starting time unspecified; 24h infusion) [n=9]	Hematologic toxicity [Neutropenia, Febrile Neutropenia, Anemia and Thrombocytopenia], Gastrointestinal toxicity [Nausea, Vomiting, Diarrhea and Stomatitis], Serum Creatinine and Potassium. (CTCAE version 4.0)	- Neutropenia: Grade II: 44.4% vs 11.1; Grade IV: 11.1% vs 44.4%.<br>- Febrile Neutropenia: Grade 3: 11.1% vs 22.2%<br>- Anemia: Grade I: 77.88% vs 66.7%; Grade II: 22.2% vs 33.3%.<br>- Vomiting: Grade I: 22.2% vs 33.3%; Grade II: 0.0% vs 11.1%<br>- Diarrhea: Grade I: 55.6% vs
	C	Day 1: Docetaxel infusion (10:30h – 11:30h) and Cisplatin (11:30h-13:30h);		

		Day 1-5: 5- Fu (13:30h; 24h infusion) [n=9]		44.4%; Grade II: 22.2% vs 33.3%.<br>- Stomatitis: Grade II: 44.4% vs 22.2%; Grade III & IV: 11.1% vs 22.2%.
Gou et al. 2018	I	Day 1-3: Infusion of Cisplatin (10:00h-22:00h); 5-Fu (22:00h – 10:00h) [n=30]	Leukocytopenia, anemia, thrombocytopenia, nausea, vomiting, blood transaminase alteration, blood/urea nitrogen alteration. (CTCAE version 3.0)	- Anemia: Grade II: 6.7% vs 16.7%.<br>- Blood transaminase: Grade I 26.7% vs 36.7%.
	C	Day 1-3: Infusion of Cisplatin (10:00h-11:00h); 5-Fu (11:00h, 24h infusion) [n=30]		
Zhang et al. 2021	I	Sinusoidal chronomodulated infusion; Cisplatin (10:00h – 22:00h with peak delivery at 16:00h) [n=75]	Leukopenia, neutropenia, anemia, thrombocytopenia, oral mucositis, gastrointestinal reactions, and liver function damage, CD3+ T count, CD3+CD4+ T count, CD3+CD8+ T count, CD4+/CD8+ ratio, CD16+CD56+ T count and CD19+ T count. (CTCAE version 3.0)	None
	C	Conventional intravenous instilling of Cisplatin (Time and duration were not reported) [n=75]		
Chen et al. 2013	I	Day 1: Paclitaxel infusion 03:00h – 05:00h and Carboplatin infusion 16:00h – 2-:00h. Day 1-5: 5-Fu continuous infusion 22:00h to 07:00h. [n=28]	Hematologic toxicity [Anemia, leukopenia, neutropenia, and thrombocytopenia], nonhematologic toxicity [Liver function abnormality, stomatitis, nausea and vomiting, diarrhea, gastrointestinal bleeding, nephropathy, fever, allergy skin toxicity, alopecia, and peripheral neurotoxicity. (WHO criteria)	- Neutropenia: Grade III-IV: 3.57% vs 14.29%<br>- Nephropathy: Grade I-II: 7.14% vs 19.05%.<br>- Fever: Grade I-II: 7.14% vs 23.81%.
	C	Day 1: Paclitaxel, Carboplatin. Day 1-5: 5-Fu. Started 09:00h – 11:00h and completed before 17:30h. [n=21]		
Ou-Yang et al. 2006	I	Day 1: Infusion of Cisplatin (10:00h-22:00h); Day 1-3: 5-Fu (22:00h – 10:00h)[n=30]	Hematologic toxicity [White blood cells, Hemoglobin, Platelets], CD3/CD19, CD3/CD4, CD3/CD8, CD3/CD 16+56 and CD4/CD8 cell levels (NCIC CTC 3.0) *All immunological parameters were tested before and after treatment within each group only.	- Hemoglobin (Grade I-IV): 30.0% vs 53.3%<br>-Platelets (Grade I-IV): 26.7% vs 56.7.
	C	Day 1: Infusion of Cisplatin (10:00h, duration unspecified); Day 1-3: 5-Fu (10:00h –22:00h) [n=30]		

Mao et al. 2015	I	Day 1: Docetaxel infusion before Cisplatin for 3-4h duration; Day 1-5: Cisplatin continuous infusion (10:00h – 22:00h); Day 1-5:<br>5-Fu (22:00h – 10:00h) [n=22]	Hematologic toxicity [Leukocytes, Neutrophils, Hemoglobin, Platelets], Gastrointestinal reactions [Nausea/Vomiting, Diarrhea and Constipation] and Oral Mucositis (CTCAE version 3.0)	None
	C	Day 1: Docetaxel infusion before Cisplatin for 3-4h duration; Day 1: Cisplatin continuous infusion *(*duration unspecified); Day 1-5: 5-Fu (24h continuous infusion) [n=23]		
Li et al. 2018	I	Day 1-7: 5-Fu (10:00h-18:00h); Day 1: Docetaxel infusion (18:30h – 19:30h) and Cisplatin infusion (19:30h – 21:30h) [n=16]	Leukopenia, neutropenia, thrombocytopenia, anemia, nausea, vomiting, and oral mucositis (WHO criteria)	None
	C	Day 1: Docetaxel infusion (1h) and Cisplatin (2h) infusion after breakfast; Day 1-7: 5- Fu infusion (micro-pump for 8h) [n=16]		
Chen et al, 2012	I	Day 1-3: Oxaliplatin (10:00h-22:00h) and 5-Fu (22:00h-10:00h) [n=23]	Nausea, vomiting, diarrhea, peripheral neuritis, leukopenia, and oral mucositis (WHO criteria)	None
	C	Day 1-3: Oxaliplatin and 5-Fu start at 9:00h per day [n=23]		
Bi et al, 2015	I	Day 1: Docetaxel (03: 30h-04: 30h); Day 1-5: Cisplatin (10:00h-22:00h); 5-Fu (22:00h-10:00h) [36]	Hematologic toxicity [Leukocytes, Neutrophils, Hemoglobin, Platelets], Gastrointestinal reactions [Nausea/Vomiting, Diarrhea and Constipation] and Oral Mucositis (CTCAE version 3.0)<br>Immune function: peripheral blood T lymphocyte subgroups (CD3+, CD4+, CD8+). CD19+, CD16+&CD56+, and CD4+/CD8+ cell ratio.	- Thrombocytopenia [Grade I-IV]: 25% vs 13.3%.<br>- Aspartate Aminotransferase (AST) increase [Grade I-IV]: 19.4% vs 30%.<br>- Elevated creatinine [Grade I-IV]: 2.7% vs 13.3%.
	C	Day 1: Docetaxel and Cisplatin infusion; Day 1-5: 5-Fu continuous infusion (120h in total) [30]		

Liu et al, 2020	I	Day 1: Docetaxel (03:30h-04:30h); Day 1-5: Cisplatin (10:00h-22:00h); 5-Fu (22:00h-10:00h) [66]	Hematologic toxicity [Anemia, leukopenia, neutropenia, and thrombocytopenia], nonhematologic toxicity [Liver function abnormality, stomatitis, nausea and vomiting, diarrhea, gastrointestinal bleeding, nephropathy, fever, allergy skin toxicity, alopecia, and peripheral neurotoxicity. (Criteria unspecified)	None.
	C	Day 1: Docetaxel and Cisplatin<br>Day 1-5: 5-Fu (120h in total) [69]		

Abbreviations: 5-Fu, Fluorouracil; CF, Citrovorum Factor; NCIC CTC, NCIC Common Toxicity Criteria; RTOG, Radiation Therapy Oncology Group; CTCAE, Common Terminology Criteria for Adverse Events; WHO, World Health Organization.

Study or Subgroup	Chronotherapy Events	Chronotherapy Total	Control Events	Control Total	Weight	Risk Ratio MH, Random, 95% CI
**Subgroup = Anemia**						
Ou-Yang and Jin 2006	0	30	2	30	0.8%	0.25 [0.01; 5.32]
Gou et al. 2018	0	30	2	30	0.8%	0.25 [0.01; 5.32]
Zhang et al. 2018	1	69	1	73	1.0%	1.06 [0.07; 16.59]
Lin et al. 2013	4	63	3	61	3.5%	1.29 [0.30; 5.53]
**Total (95% CI)**		**192**		**194**	**6.0%**	**0.81 [0.22; 2.98]**
Heterogeneity: $Tau^2 = 0$; $Chi^2 = 1.57$, df = 3 (P = 0.67); $I^2 = 0\%$						
**Subgroup = Leukopenia**						
Ou-Yang and Jin 2006	0	30	3	30	0.8%	0.17 [0.01; 3.19]
Gou et al. 2018	0	30	3	30	0.8%	0.17 [0.01; 3.19]
Chen et al. 2012	0	23	1	23	0.7%	0.50 [0.02; 14.18]
Bi et al. 2015	6	36	9	30	8.8%	0.56 [0.22; 1.38]
Mao et al. 2015	4	22	7	23	6.3%	0.60 [0.20; 1.76]
Lin et al. 2013	3	63	4	61	3.5%	0.73 [0.17; 3.11]
Zhang et al. 2018	14	69	16	73	18.1%	0.93 [0.49; 1.75]
**Total (95% CI)**		**273**		**270**	**39.0%**	**0.69 [0.47; 1.01]**
Heterogeneity: $Tau^2 = 0$; $Chi^2 = 2.92$, df = 6 (P = 0.82); $I^2 = 0\%$						
**Subgroup = Neutropenia**						
Li et al. 2018	0	16	8	16	1.0%	0.06 [0.00; 1.00]
Tsuchiya et al. 2018	5	9	8	9	18.6%	0.63 [0.33; 1.17]
Zhang et al. 2018	5	69	8	73	6.4%	0.66 [0.23; 1.92]
Bi et al. 2015	11	36	13	30	17.9%	0.71 [0.37; 1.34]
Lin et al. 2013	3	63	4	61	3.5%	0.73 [0.17; 3.11]
Mao et al. 2015	5	22	7	23	7.5%	0.75 [0.28; 2.00]
**Total (95% CI)**		**215**		**212**	**55.0%**	**0.65 [0.45; 0.94]**
Heterogeneity: $Tau^2 = 0$; $Chi^2 = 2.92$, df = 5 (P = 0.71); $I^2 = 0\%$						
**Total (95% CI)**		**680**		**676**	**100.0%**	**0.68 [0.55; 0.83]**
Heterogeneity: $Tau^2 = 0$; $Chi^2 = 7.57$, df = 16 (P = 0.96); $I^2 = 0\%$						
Test for subgroup differences: $Chi^2 = 0.30$, df = 2 (P = 0.86)						

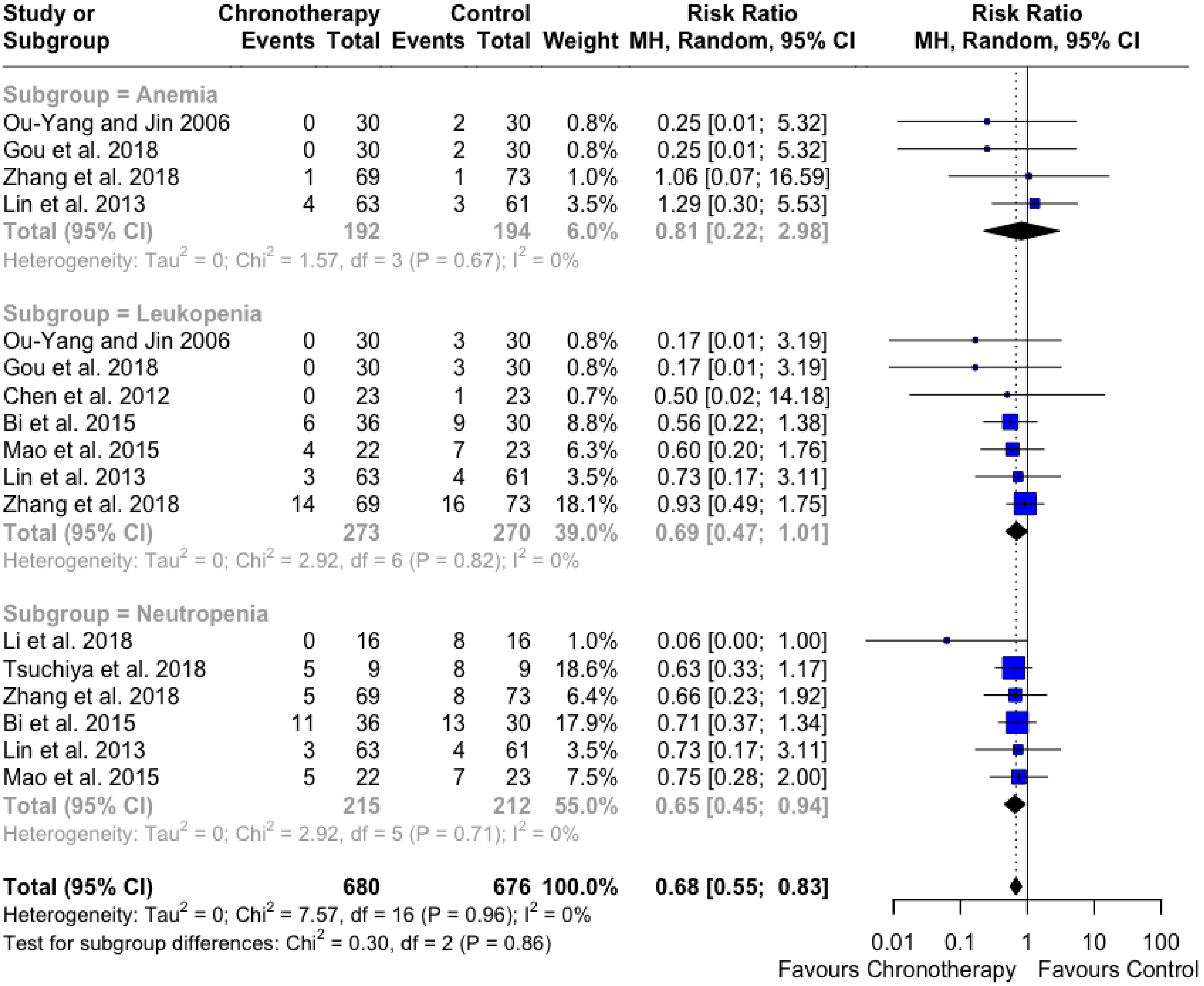

Supplemental Figure 1. Effect of chrono-chemotherapy on haematological toxicity and adverse events.

Study or Subgroup	Chronotherapy Events	Chronotherapy Total	Control Events	Control Total	Weight	Risk Ratio MH, Random, 95% CI
**Subgroup = Diarrhea**						
Bi et al. 2015	0	36	3	30	2.5%	0.14 [0.01; 2.67]
Chen et al. 2012	0	23	3	23	2.5%	0.17 [0.01; 3.15]
Tsuchiya et al. 2018	1	9	1	9	3.2%	1.00 [0.07; 13.64]
Mao et al. 2015	3	22	1	23	4.5%	3.14 [0.35; 27.92]
**Total (95% CI)**		**90**		**85**	**12.7%**	**0.66 [0.06; 7.52]**
Heterogeneity: $Tau^2 = 0.5248$; $Chi^2 = 3.95$, df = 3 (P = 0.27); $I^2 = 24\%$						
**Subgroup = Nausea and Vomiting**						
Zhang et al. 2018	0	69	10	73	2.7%	0.05 [0.00; 0.89]
Bi et al. 2015	0	36	4	30	2.6%	0.10 [0.01; 1.89]
Li et al. 2018	3	16	10	16	18.3%	0.30 [0.10; 0.89]
Tsuchiya et al. 2018	2	9	6	9	12.7%	0.33 [0.09; 1.23]
Mao et al. 2015	1	22	2	23	4.0%	0.52 [0.05; 5.36]
Lin et al. 2013	3	63	5	61	11.3%	0.58 [0.15; 2.33]
**Total (95% CI)**		**215**		**212**	**51.6%**	**0.32 [0.17; 0.62]**
Heterogeneity: $Tau^2 = 0$; $Chi^2 = 3.04$, df = 5 (P = 0.69); $I^2 = 0\%$						
**Subgroup = Stomatitis**						
Zhang et al. 2018	0	69	11	73	2.7%	0.05 [0.00; 0.80]
Chen et al. 2012	0	23	1	23	1.9%	0.50 [0.02; 14.18]
Tsuchiya et al. 2018	2	9	4	9	10.7%	0.50 [0.12; 2.08]
Lin et al. 2013	5	63	9	61	20.3%	0.54 [0.19; 1.51]
**Total (95% CI)**		**164**		**166**	**35.6%**	**0.44 [0.14; 1.40]**
Heterogeneity: $Tau^2 = 0$; $Chi^2 = 2.56$, df = 3 (P = 0.47); $I^2 = 0\%$						
**Total (95% CI)**		**469**		**463**	**100.0%**	**0.40 [0.25; 0.63]**
Heterogeneity: $Tau^2 = 0$; $Chi^2 = 10.79$, df = 13 (P = 0.63); $I^2 = 0\%$						
Test for subgroup differences: $Chi^2 = 1.08$, df = 2 (P = 0.58)						

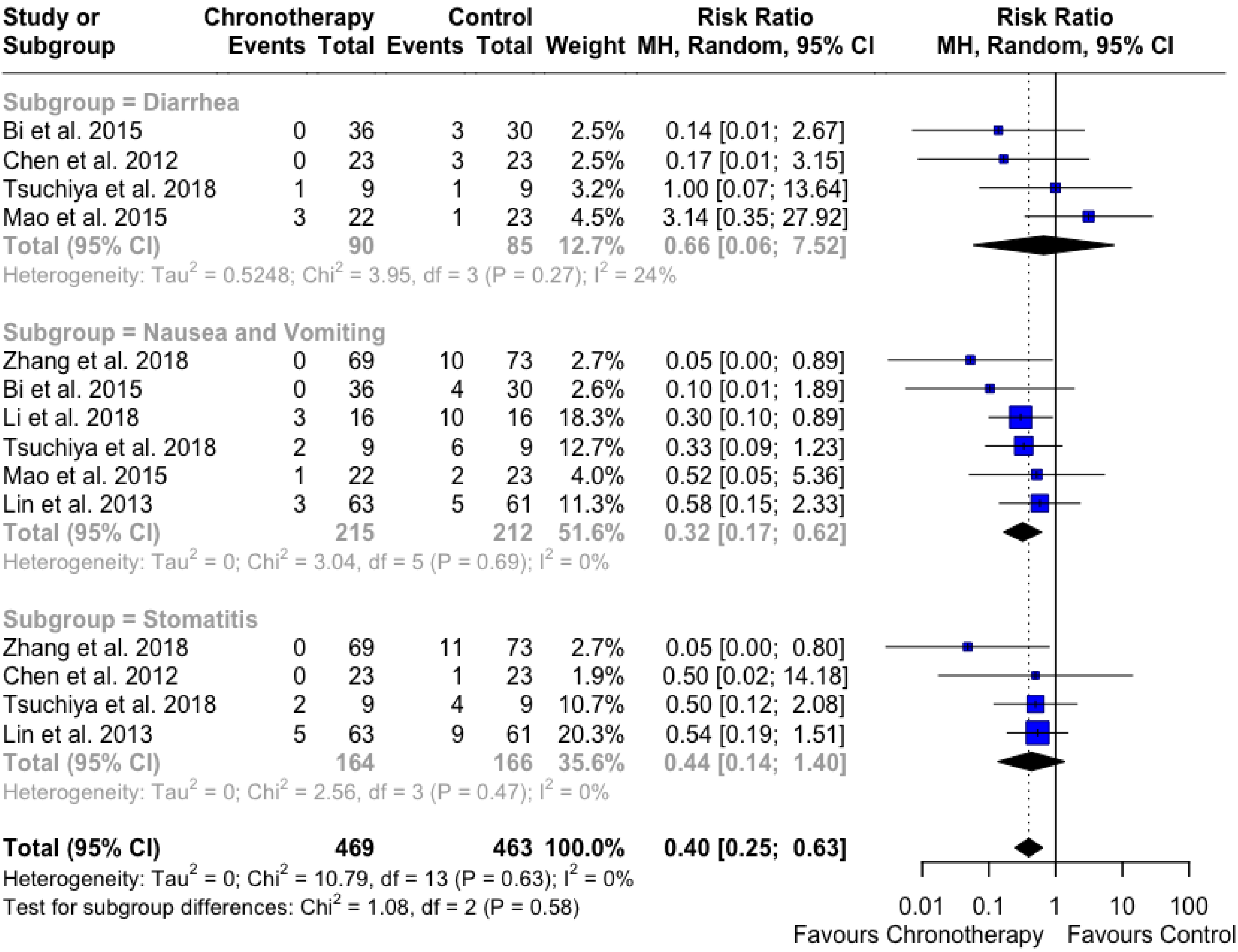

Supplemental Figure 2. Effect of chrono-chemotherapy on gastrointestinal toxicity and adverse events.
